# A prospective evaluation of the fourth national Be Clear on Cancer ‘Blood in Pee’ campaign in England

**DOI:** 10.1111/ecc.13606

**Published:** 2022-05-15

**Authors:** Samuel William David Merriel, Susan Ball, Chloe Jayne Bright, Vivian Mak, Carolynn Gildea, Lizz Paley, Chris Hyde, William Hamilton, Lucy Elliss‐Brookes

**Affiliations:** ^1^ Institute of Health Research, University of Exeter Medical School University of Exeter Exeter UK; ^2^ NIHR ARC South West Peninsula (PenARC), University of Exeter Medical School University of Exeter Exeter UK; ^3^ National Cancer Registration and Analysis Service Public Health England London UK; ^4^ NHS Digital Leeds UK

**Keywords:** awareness, early diagnosis, haematuria, kidney neoplasms, public health, urinary bladder neoplasms

## Abstract

**Objective:**

To assess the impact of the fourth Be Clear on Cancer (BCoC) ‘Blood in Pee’ (BiP) campaign (July to September 2018) on bladder and kidney cancer symptom awareness and outcomes in England.

**Methods:**

In this uncontrolled before and after study, symptom awareness and reported barriers to GP attendance were assessed using panel and one‐to‐one interviews. The Health Improvement Network (THIN), National Cancer Registration and Analysis Service (NCRAS) and NHS Cancer Waiting Times (CWT) data were analysed to assess the impact on GP attendances, urgent cancer referrals, cancer diagnoses and 1‐year survival. Analyses used Poisson, negative binomial and Cox regression.

**Results:**

Symptom awareness and intention to consult a GP after one episode of haematuria increased following the campaign. GP attendance with haematuria (rate ratio (RR) 1.17, 95% confidence interval (CI): 1.07–1.28) and urgent cancer referrals (RR 1.18 95% CI: 1.08–1.28) increased following the campaign. Early‐stage diagnoses increased for bladder cancer (difference in percentage 2.8%, 95% CI: −0.2%–5.8%), but not for kidney cancer (difference −0.6%, 95% CI: −3.2%–2.1%).

**Conclusions:**

The fourth BCoC BiP campaign appears to have been effective in increasing bladder cancer symptom awareness and GP attendances, although long‐term impacts are unclear.

## 1 INTRODUCTION

Urological cancers are amongst the most common cancers diagnosed in England. Whilst prostate cancer predominates within urological cancers, there were almost 20,000 new cases of urological cancer from other sites, including bladder, kidney and ureteric cancers, in 2018(ONS, 2020a). Age‐standardised bladder cancer incidence between 1993 and 2017 fell by 41.4% across the United Kingdom, more markedly in males than females, probably due to lower smoking rates and reduced occupational exposure to carcinogens. Males (27 cases per 100,000) still have a much higher incidence rate than females (eight per 100,000) (ONS, 2020a). Age‐standardised kidney cancer incidence in the United Kingdom increased by 86.4% from 1993 to 2017, more so amongst females. Males have a similar incidence of kidney cancer (28 cases per 100,000) as for bladder cancer, although females have a higher incidence of kidney cancer (15 per 100,000) (Cancer Research UK, 2020). Age‐standardised 5‐year survival is similar for bladder (52.6%) and kidney (63.8%) cancers (ONS, 2020a, 2020b).

Haematuria, or blood in the urine, is a common high‐risk symptom of urological cancer, particularly bladder cancer. Haematuria can be visible to patients (macroscopic) or only detectable with a urine dipstick test (microscopic). Macroscopic haematuria is the most common symptom that patients with bladder cancer report to primary care prior to diagnosis, accounting for over half of all presentations (Shephard et al., 2012). Macroscopic haematuria is also the most common symptom of kidney cancer (reported by 17.7%), with other common presenting symptoms being much less specific (i.e. back pain, abdominal pain and fatigue) (Shephard et al., 2013). In England in 2017, 69.4% of patients with a new diagnosis of bladder cancer and 58.9% of new kidney cancer cases were diagnosed following a referral from their GP after reporting symptoms (National Cancer Intelligence Network, 2020).

Cancer symptom awareness starts with a person recognising a bodily change and then appraising the symptom to decide on seriousness. Individuals who are worried about their symptoms then consult a healthcare professional, most often a GP in the United Kingdom (Walter et al., 2012). Prompt presentation with potential cancer symptoms is important for the early diagnosis of cancer; however, 25.8% of patients with bladder cancer and 30.1% of kidney cancer patients with symptoms took longer than 14 days to present to their GP (Keeble et al., 2014). Cancer symptom awareness in the United Kingdom is lower amongst individuals under 35 or over 74 years of age, males, single people, the unemployed and those from areas of high deprivation (Forbes et al., 2014; McCutchan et al., 2015; Niksic et al., 2015). Lower cancer symptom awareness and higher perceived barriers to accessing healthcare for symptomatic patients are both associated with lower cancer survival (Forbes et al., 2015; Niksic et al., 2016). Fear and fatalistic beliefs relating to cancer can delay presentation with such symptoms, particularly in deprived populations (McCutchan et al., 2015; Niksic et al., 2015).

The Be Clear on Cancer (BCoC) programme, led by Public Health England (PHE), delivers campaigns that aim to improve the early diagnosis of cancer by raising public awareness of symptoms and signs of cancer and encouraging people to attend their GP without delay (Public Health England, 2020). To date there have been four BCoC ‘Blood in Pee’ (BiP) campaigns focusing on raising awareness of symptoms of bladder or kidney cancer. These campaigns were delivered through a variety of media in England, predominantly targeting those aged over 50 years of age and those from lower socio‐economic groups. For example, TV advertising was bought in programmes popular with those aged over 50 from lower socio‐economic groups. An evaluation report by PHE of the pilot campaigns and the first three national BCoC BiP campaigns has been published online (Kockelbergh, 2020), which showed mixed results. There was an increase in GP presentations, referrals and cancers diagnosed, but no clear impact on stage at diagnosis or 1‐year survival. The aim of this study was to assess independently the impact of the fourth BCoC ‘BiP’ campaign on bladder and kidney cancer symptom awareness and outcomes. A secondary aim was to explore the long‐term trends in bladder and kidney cancer diagnoses in the context of the four BCoC BiP campaigns.

## 2 METHODS

Reporting of this study has been guided by the Strengthening The Reporting of Observational Studies in Epidemiology (STROBE) statement (von Elm et al., 2007). A completed STROBE checklist can be found in the Supporting Information.

### 2.1 Campaign overview

The fourth national BiP campaign ran from 19 July 2018 to 16 September 2018 in England. The aim of the campaign was to raise awareness of symptoms of bladder and kidney cancer and encourage patients with these symptoms to present to their GP to facilitate earlier diagnosis. The campaign ran under the ‘Be Clear on Cancer’ brand with the core message:

If you notice blood in your pee, even if it's ‘just the once’, tell your doctor.

Channels used to deliver the campaign included TV, radio, press and out of home (in public toilets in shopping centres, bars and motorway service stations) advertising; digital advertising through Facebook and search engines; public relations activities by PHE; and working with partners including cancer charities and community organisations. There was also activity targeted at those aged 50 and over from Black and South Asian audiences. This included TV, radio and public toilet advertising; public relations; partnership activity; and outreach events. Further details on the campaign messaging, advertisements and resources used can be found on the PHE Campaign Resource Centre (Public Health England, 2020). Previous BCOC BiP campaigns ran from October to November 2013 (first campaign), October to November 2014 (second campaign) and February to March 2016 (third campaign).

### 2.2 Data collection and variables

This study utilised an uncontrolled before and after study design. Data were analysed for a range of variables (herein ‘metrics’) spanning the patient pathway for bladder and kidney cancer diagnosis. The ‘analysis period’ was the time period during and shortly after the campaign and varied for each metric to take into account when an impact of the campaign would be expected (i.e. attendance at a GP is expected to occur more quickly than a diagnosis of cancer). The comparison period was the same time period during the previous year, 2017, thereby reducing risk of seasonal effects on metrics (see Table 1). More details on defining the analysis and comparison periods for each metric have been published on PHE's website (http://www.ncin.org.uk/BCOC) and can be viewed in Table 1.

**TABLE 1:** Analysis and comparison periods used in the analysis for each metric

Metric	Breakdown	Comparison period	Analysis period
Symptom awareness	N/A	22 June 2018 to 1 July 2018	21 to 30 September 2018
GP attendances	Week	24 July 2017 to 1 October 2017	23 July 2018 to 30 September 2018
Urgent cancer referrals	Month	July 2017 to October 2017	July 2018 to October 2018
Cancer diagnosed from urgent cancer referral	Month	July 2017 to October 2017	July 2018 to October 2018
Emergency cancer diagnoses	Month	July 2017 to October 2017	July 2018 to October 2018
Cancer diagnoses in CWT database	Month	August 2017 to November 2017	August 2018 to November 2018
Cancers diagnosed	Week	7 August 2017 to 19 November 2017	6 August 2018 to 18 November 2018
Early stage at diagnosis	Week	7 August 2017 to 19 November 2017	6 August 2018 to 18 November 2018
Diagnostics in secondary care	Month	August 2017 to November 2017	August 2018 to November 2018
Survival	N/A	7 August 2017 to 19 November 2017	6 August 2018 to 18 November 2018

#### 2.2.1 Symptom awareness and barriers to seeking care

Measurements of symptom awareness and barriers to attending their GP from the fourth BCoC BiP campaign were undertaken using online questionnaires for those aged 50–69 years of age and face‐to‐face interviews at home for those aged over 70. Participants were recruited via a market and social research agency (Kantar Ltd) through face‐to‐face approaches to members of the public in public spaces and shopping centres and online panels, employing quotas to obtain a balance of age, gender, socio‐economic status ([SES] using the National Readership Survey[NRS] classification—http://www.nrs.co.uk/) and geographic region. Questionnaires were piloted and developed in previous BCoC campaign evaluations for other cancer types (Lai et al., 2020) and adapted for the fourth BCoC BiP campaign (see Supporting Information). Data collection was performed using questionnaires delivered online or in‐person using tablet computers. Pre‐campaign data collection occurred from 22 June to 1 July 2018, and post‐campaign data collection occurred between 21 and 30 September 2018.

#### 2.2.2 GP attendances

Data on GP attendances for visible blood in pee (macroscopic haematuria) were sourced from The Health Improvement Network (THIN) database (THIN, 2020). This is a primary care database containing anonymised copies of GP records from approximately 6% of the UK population. Consultation data were grouped into weeks and adjusted to account for bank holidays. Information on the number of GP practices submitting data each week (which decreased from 177 to 116 practices over the period considered) was also collected to enable the calculation of the average number of attendances per practice per week.

#### 2.2.3 Urgent cancer referrals for suspected urological cancer (two‐week‐wait [TWW] referrals) and cancers diagnosed from an urgent cancer referral

Data on urgent cancer referrals for suspected urological cancer and cancer diagnoses that resulted from an urgent cancer referral were collected from the National Cancer Waiting Times Monitoring Data Set, provided by NHS England. The data were grouped according to the month the patient was first seen. Cancers were defined using ICD‐10 as bladder (C67), kidney and urinary tract (C64–C66 and C68) and urological (including prostate) (C60–C61 and C63–68).

#### 2.2.4 Emergency cancer diagnoses

Monthly data on the number of emergency cancer diagnoses were sourced from the Hospital Episodes Statistics (HES) Admitted Patient Care data linked to cancer registration data held by the National Cancer Registration and Analysis Service (NCRAS) (NCRAS, 2020b) using methodology outlined in NCRAS Official Statistics Emergency Presentation metric (NCRAS, 2020a). Cancers were defined using ICD‐10 as bladder (C67), kidney and urinary tract (C64–C66 and C68).

#### 2.2.5 Cancer diagnoses in the Cancer Waiting Times (CWT) database

Data on the number of urological cancer diagnoses in the Cancer Waiting Times (CWT) database from all routes to diagnosis were sourced from the National Cancer Waiting Times Monitoring Data Set, provided by NHS England. The data were grouped according to the month the patient was first treated. Cancers were defined using ICD‐10 as bladder (C67), kidney and urinary tract (C64–C66 and C68) and urological (including prostate) (C60–C61 and C63–68).

#### 2.2.6 Cancers diagnosed and stage at diagnosis

Data on the number of bladder and kidney cancers diagnosed and the stage at diagnosis were sourced from the National Cancer Registration Dataset collected by NCRAS (Henson et al., 2020). The data were again grouped into weeks and adjusted to account for bank holidays. Cancers were defined as malignant bladder cancer (ICD‐10 C67), kidney and urinary tract (ICD‐10 C64–C66 and C68), carcinoma in situ of bladder (ICD‐10 D09.0) and non‐invasive papillary carcinoma (pTa) of the bladder (ICD‐10 D41.4).

The stage at diagnosis metric was restricted to malignant cancers only. For bladder cancer, early stage was defined as TNM Stage 1 only, because TNM Stage 2 bladder cancer has grown into the muscle layer of the bladder. For kidney cancer, early stage was defined as TNM Stage 1 or 2. The denominator included cases with a valid stage recorded.

#### 2.2.7 Diagnostic activity in secondary care

Data on the monthly number of ultrasounds, magnetic resonance imaging (MRI) and computerised tomography (CT) scans were obtained from the Diagnostic Imaging Dataset (DID) held on NHS Digital's iView system (https://iview.hscic.gov.uk/Home/About).

#### 2.2.8 Survival

Patients diagnosed with bladder and kidney cancer (in the National Cancer Registration Dataset) were traced using the Patient Demographic Service (held by NHS Digital) to obtain date of death or last follow‐up.

### 2.3 Statistical analysis

For survey questions relating to symptom awareness and intended action, the percentage of respondents who chose the response option, or set of options, that were the target of the campaign (i.e. indicated symptom awareness and correct intended actions) were compared between surveys conducted in the periods before and after the campaign. Comparisons used the two sample test of proportions. Responses were weighted using the Random Iterative Method (RIM) according to age, gender and SES, and a continuity correction was applied.

Metrics which included either weekly or monthly counts (all except early stage at diagnosis and survival) were analysed using Poisson regression or negative binomial regression with one explanatory variable coded as 0 or 1 for the comparison and analysis period. Results are presented as the total count in each period, plus the estimated rate ratio (RR) (analysis period relative to the comparison period) with the 95% CI and *p*‐value. For GP attendances, the number of GP practices contributing data into THIN each week was added as an offset to the model; therefore, the results are presented as the count per practice. For the early stage at diagnosis metric, counts were aggregated, and percentages calculated for the comparison and analysis period. The absolute percentage change between the two periods (analysis–comparison period) was calculated, and a *p*‐value reported from a two‐sample test of proportions. Survival data were compared using Cox regression, with time to death or end of follow‐up (1 year) as the outcome and reported as the estimated hazard ratio (analysis period relative to comparison period) with the 95% CI and *p*‐value. One‐year age specific net survival was calculated for each period using methodology outlined in the Office for National Statistics: Cancer Survival Bulletins (ONS, 2021).

Analyses were based on people of all ages, with sensitivity analyses examining the effect of age and gender. Trends in metrics over time were inspected using visual displays, with data covering 2012–2018. All statistical tests were two sided, with no adjustment for multiple testing. Analyses were conducted in Stata 16 (StataCorp. 2019. *Stata Statistical Software: Release 16*. College Station, TX: StataCorp LLC) and R (R core Team, 2019).

### 2.4 Ethics statement

All data used in this study were acquired from external sources, none of which was identifiable. No primary data collection or participant recruitment was undertaken by the study team. Participants in the symptom awareness survey were recruited from a cohort of a pre‐existing market and social research agency (Kantar Ltd), and all had given prior consent to Kantar to be approached for such surveys and to have their responses shared with third parties. All remaining data were acquired from publically owned, anonymised datasets. As such, ethical approval for this study was not sought.

## 3 RESULTS

### 3.1 Symptom awareness

876 participants were recruited for the symptom awareness survey following the campaign (analysis period), and 820 were recruited prior to the campaign (comparison period), for a total of 1696 survey participants. See Table 2 for demographic characteristics. There was an increase in the percentage of respondents correctly identifying that seeing blood in their pee could be a symptom of cancer following the campaign (21.0% difference [post–pre], 95% CI: 16.2%–25.7%). Confidence in knowing the potential cause of blood in pee also increased (12.6% difference [post–pre], 95% CI: 7.8%–17.5%). There was a 5.9% (95% CI: 1.3%–10.6%) increase in the number of patients that reported they would visit their GP after seeing blood in their pee once. There was no clear change in participant's attitudes towards the importance of early diagnosis for treatment or survival outcomes following the campaign. See Table 3 for further results.

**TABLE 2:** Symptom awareness survey sample characteristics

Characteristic	Comparison period *n* (%)	Analysis period *n* (%)
Total participants (*n* = 1696)	876 (51.7%)	820 (48.3%)
Age (years)		
50–69 (online)	550 (62.8%)	550 (67.1%)
70+ (face‐to‐face)	326 (37.2%)	270 (33.9%)
Male	420 (47.9%)	400 (48.8%)
Female	456 (52.1%)	420 (51.2%)
National Readership Survey (NRS) demographics		
ABC1	449 (51.3%)	458 (55.6%)
C2DE	427 (48.7%)	362 (44.4%)

**TABLE 3:** RIM‐weighted responses to cancer symptom awareness questionnaire and barriers to presentation

	Comparison period *N* = 876	Analysis period *N* = 820	Absolute difference in percentage (95% CI)	*p*‐value
**What, if anything, would you be likely to do if your pee was a different colour than you had seen before?**				
Visit your GP, *n* (%)	237 (27.1)	249 (30.4)	3.3 (−1.1 to 7.7)	0.15
**What, if anything, would you be likely to do if you noticed blood in your pee just once?**				
Visit your GP, *n* (%)	411 (46.9)	398 (48.5)	1.6 (−3.3 to 6.5)	0.54
**After how many times of seeing blood in your pee, would you go and visit your GP?**				
Once, *n* (%)	275 (31.4)	306 (37.3)	5.9 (1.3 to 10.6)	0.01
**If a person sees blood in their pee, what do you think it could be a symptom of?**				
Cancer, *n* (%)	296 (33.8)	449 (54.8)	21.0 (16.2 to 25.7)	<0.001
**How confident are you that you know what blood in pee could be a sign of?**				
Confident^a^, *n* (%)	369 (42.1)	449 (54.8)	12.6 (7.8 to 17.5)	<0.001
**How much do you agree or disagree with the statement: Blood in pee could be a sign of cancer**				
Agree^b^, *n* (%)	660 (75.3)	690 (84.2)	8.8 (4.9 to 12.7)	<0.001
**How much do you agree or disagree with the statements: option analysed for each = *disagree*** ^c^				
**I wouldn't worry about having blood in my pee if I had no other symptoms, *n* (%)**	644 (73.5)	614 (74.9)	1.4 (−2.9 to 5.7)	0.56
**I would only do something about blood in my pee if I saw it several times, *n* (%)**	310 (35.4)	339 (41.3)	5.9 (1.2 to 10.7)	0.01
**When thinking about visiting a GP as soon as possible the first time you saw blood in your pee, would you say …**				
I would definitely, or very likely, do this^d^, *n* (%)	402 (45.9)	443 (54.0)	8.1 (3.3 to 13.0)	<0.001
**Having blood in your pee is one of the symptoms of kidney and bladder cancers … Were you aware of this before today?**				
Yes, *n* (%)	501 (57.2)	552 (67.3)	10.1 (5.4 to 14.8)	<0.001
**How much do you agree or disagree with the statement: If kidney or bladder cancer is diagnosed early, it is more likely to be treatable**				
Agree^b^, *n* (%)	788 (89.9)	727 (88.7)	−1.3 (−4.4 to 1.8)	0.43
**How much do you agree or disagree with the statement: Going to my GP early with a symptom of kidney or bladder cancer makes no difference to my chances of surviving cancer**				
Disagree^c^, *n* (%)	656 (74.9)	599 (73.1)	−1.8 (−6.1 to 2.5)	0.42

^a^ Includes responses of ‘very confident’ and ‘fairly confident’, out of possible responses ‘very confident’, ‘fairly confident’, ‘not very confident’, ‘not at all confident, I have just guessed’ and ‘don't know’.

^b^ Includes responses of ‘strongly agree’ and ‘agree’, out of possible responses ‘strongly agree’, ‘agree’, ‘disagree’, ‘strongly disagree’ and ‘don't know’.

^c^ Includes responses of ‘disagree’ and ‘strongly disagree’, out of possible responses ‘strongly agree’, ‘agree’, ‘disagree’, ‘strongly disagree’ and ‘don't know’.

^d^ Includes responses ‘10’ (I definitely would do this), ‘9’ and ‘8’, out of possible responses 0 (I definitely wouldn't do this) to 10.

### 3.2 GP attendances

**FIGURE 1:**
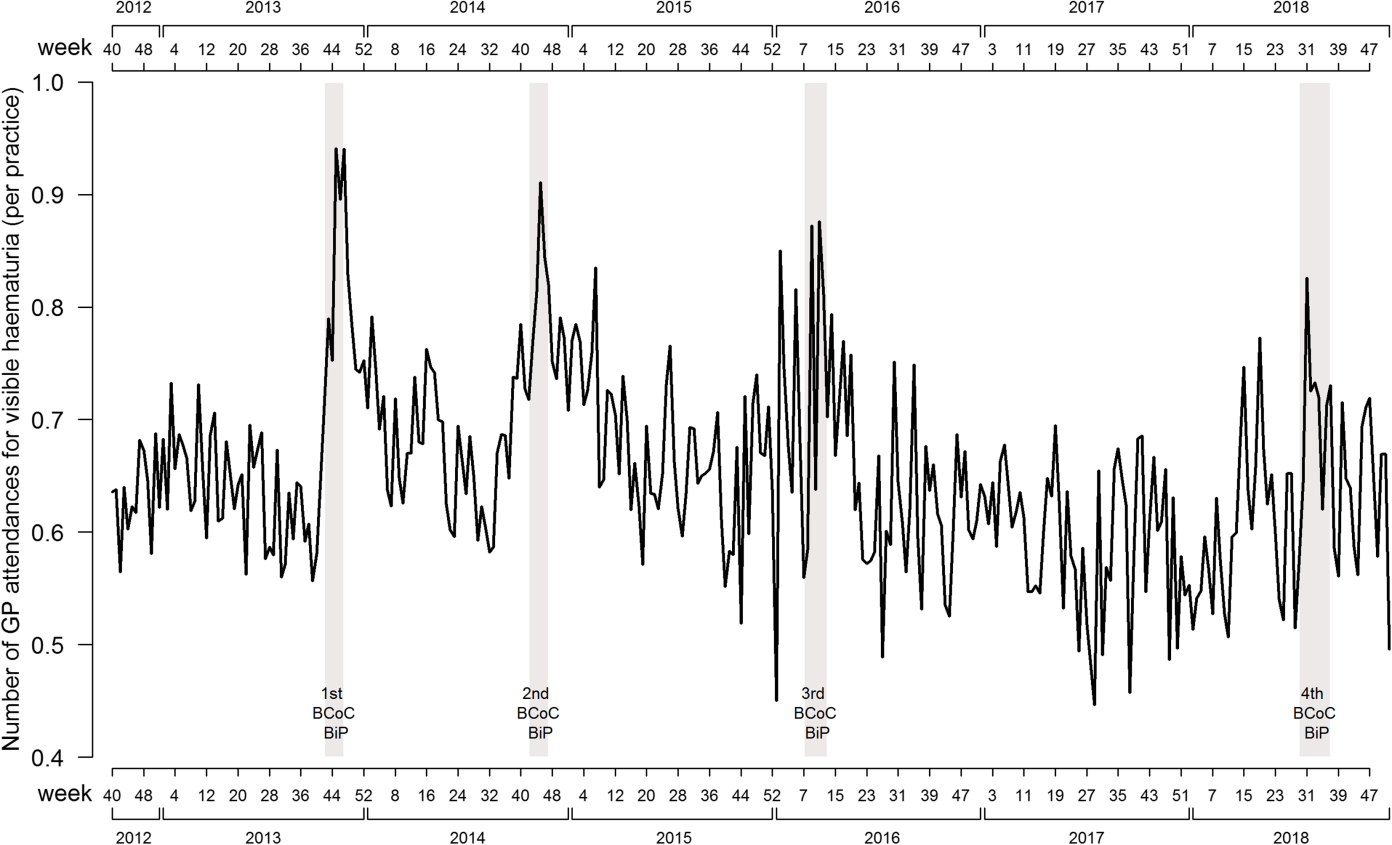
Trend line for GP presentations with haematuria from October 2012 to December 2018

The rate of GP attendances for haematuria was 17% higher during the analysis period relative to the comparison period (estimated RR 1.17, 95% confidence interval (CI): 1.07 to 1.28) (see Table 4). This increase was driven by males, for whom there was a 24% increase during the analysis period compared to the comparison period (RR 1.24, 95% CI: 1.11–1.40) (see Table S2). Figure 1 shows the long‐term trends for GP presentations with haematuria, which demonstrates an increase during each of the BCoC BiP campaigns including the fourth campaign. Following these peaks, presentations with haematuria appear to return to pre‐campaign levels.

**TABLE 4:** Results of metrics—all ages

Metric	Type of symptom/referral/cancer	Comparison period	Analysis period	Statistic	Estimate (95% CI)	p‐value
GP attendances	Blood in pee	0.59 attendances per practice per week	0.69 attendances per practice per week	Rate ratio	1.17 (1.07–1.28)	<0.001
Urgent cancer referrals	Suspected urological cancer	63,257	74,422	Rate ratio	1.18 (1.08–1.28)	<0.001
Cancer diagnosed from urgent cancer referral for suspected urological cancer	Bladder	1934	1950	Rate ratio	1.01 (0.92–1.11)	0.86
	Kidney and urinary tract	1006	1124	Rate ratio	1.12 (0.98–1.27)	0.09
	Urological cancer (including prostate)	10,663	12,024	Rate ratio	1.13 (1.03–1.24)	0.01
Emergency cancer diagnoses	Bladder	8.6% (476 of 5510)	7.6% (423 of 5598)	Difference in percentage	−1.1% (−2.1% to −0.1%)	0.04
	Kidney and unspecified urinary organ	16.3% (424 of 2608)	16.5% (441 of 2,673)	Difference in percentage	0.2% (−1.8%–2.2%)	0.81
Cancer diagnoses in CWT database	Bladder	3019	2985	Rate ratio	0.99 (0.92–1.07)	0.77
	Kidney and urinary tract	2926	2952	Rate ratio	1.01 (0.92–1.11)	0.86
	Urological cancer (including prostate)	18,193	20,646	Rate ratio	1.13 (1.06–1.21)	<0.001
Cancer diagnoses in National Cancer Registration Dataset	Malignant bladder	2528	2448.75	Rate ratio	0.97 (0.92–1.02)	0.26
	Bladder carcinoma in situ	2389.75	2653.5	Rate ratio	1.11 (1.05–1.17)	<0.001
	Kidney and urinary tract	3270.5	3271.5	Rate ratio	1.00 (0.95–1.05)	0.99
	pTa	247	246	Rate ratio	1.00 (0.83–1.19)	0.96
Early stage at diagnosis	Malignant bladder	46.3% (1021.5 of 2208.25 staged cases)	49.1% (1016.5 of 2072.25 staged cases)	Difference in percentage	2.8% (−0.2%–5.8%)	0.07
	Kidney and urinary tract	54.4% (1482 of 2722.25 staged cases)	53.9% (1471.75 of 2732.5 staged cases)	Difference in percentage	−0.6% (−3.2%–2.1%)	0.67
Diagnostics in secondary care	Ultrasounds, MRIs and CT scans	460,520	506,920	Rate ratio	1.10 (1.06–1.14)	<0.001
1 year survival	Bladder	70.5%	70.5%	Hazard ratio	0.93 (0.83–0.99)	0.02
	Kidney	78.6%	79.6%	Hazard ratio	0.98 (0.91–1.05)	0.51

### 3.3 Urgent cancer referrals for suspected urological cancer and cancers diagnosed from an urgent cancer referral

**FIGURE 2:**
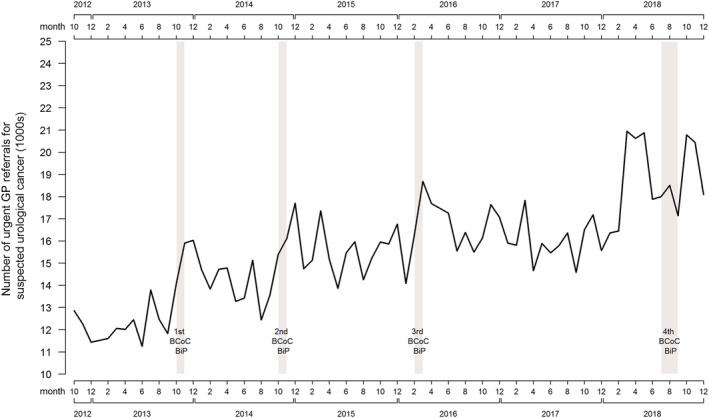
Trend line for urgent cancer referrals for suspected urological cancer from October 2012 to December 2018

**FIGURE 3:**
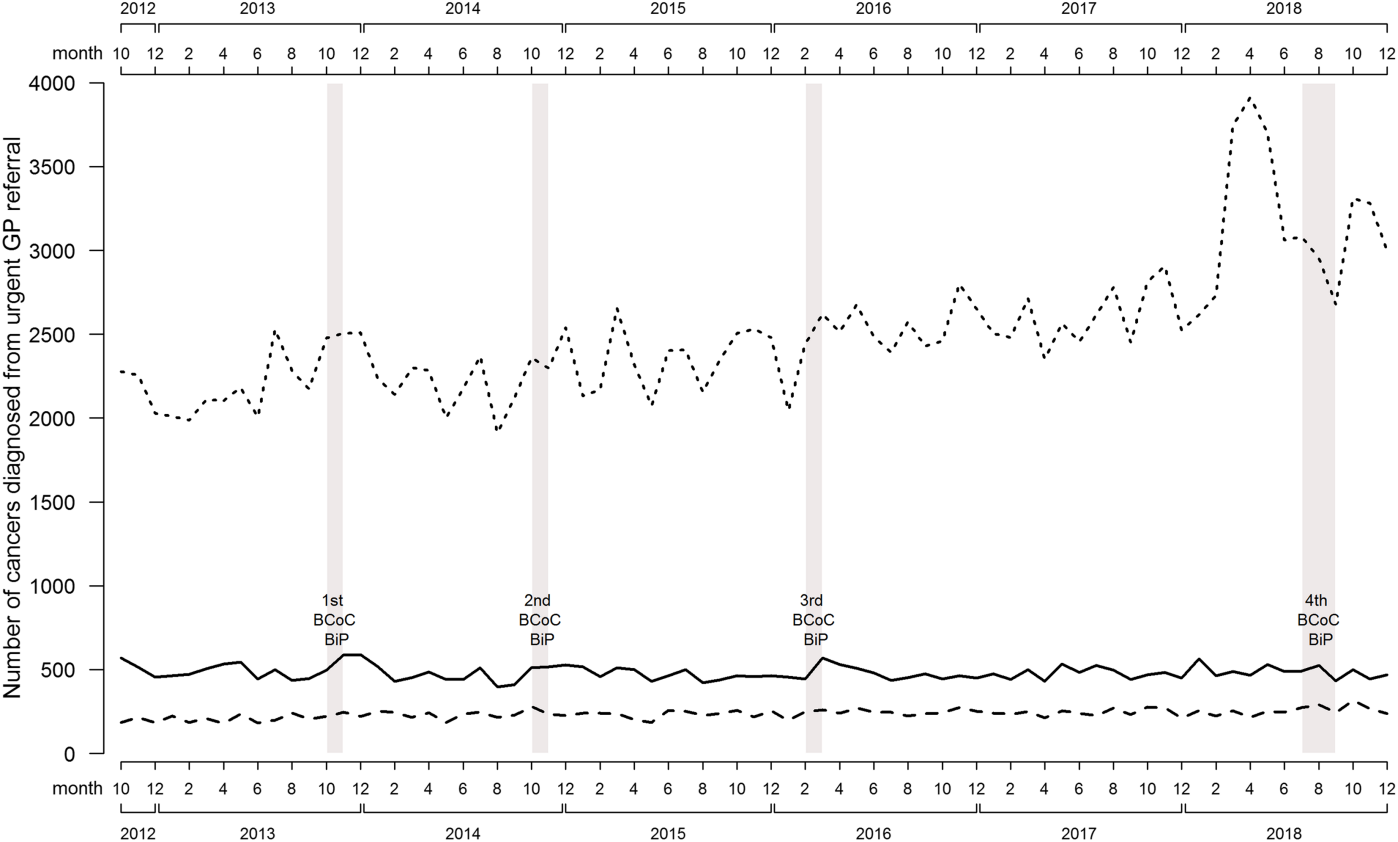
Trend line for cancers diagnosed from urgent cancer referrals between October 2012 and December 2018 (solid line = bladder; dashed line = kidney and urinary tract; dotted line = urological [including prostate])

Urgent cancer referrals for suspected urological cancer were higher during the analysis period relative to the comparison period (RR 1.18, 95% CI: 1.08–1.28). The long‐term trend of urgent cancer referrals for suspected urological cancer is upwards. Even allowing for this trend, Figure 2 shows sharper increases in referrals during and after the campaigns. There was also a marked increase in referrals immediately prior to the fourth campaign in Months 3–5 of 2018. The numbers of kidney and urinary tract cancers diagnosed from an urgent cancer referral for suspected urological cancer increased during the analysis period relative to the comparison period; however, this was not statistically significant (RR 1.12, 95% CI: 0.98–1.27). Bladder cancer diagnoses from urgent referral was similar in the analysis and comparison periods (RR 1.01, 95% CI: 0.92–1.11), although longer‐term trends suggest an increase in bladder cancer diagnoses from the first three campaigns (see Figure 3). The numbers of urological cancers (including prostate and rarer urological cancer types) diagnosed from urgent referral were 13% higher in the analysis period, relative to the comparison period (RR 1.13, 95% CI: 1.03–1.24), with a much more pronounced increase in urological cancer diagnoses for Months 3–5 of 2018 (a few months prior to the fourth campaign).

### 3.4 Emergency cancer diagnoses

There was some indication of a reduction in the percentage of bladder cancer diagnoses referred by a GP as an emergency (difference [analysis − comparison] −1.1%, 95% CI: −2.1% to −0.1%), but no change for kidney cancers between the analysis and comparison periods. These findings are in the context of a long‐term trend of reducing emergency diagnosis of urological cancers (see Figure S1).

### 3.5 Cancer diagnoses in the CWT database

There was no evidence of a difference in bladder cancer and kidney and urinary tract cancer diagnoses in the CWT database between the analysis and comparison period (bladder: RR 0.99, 95% CI: 0.92–1.07; kidney and urinary tract: RR 1.01, 95% CI: 0.92–1.11). There was an increase in urological cancer diagnoses in the CWT database in the analysis period, relative to the comparison period (RR 1.13, 95% CI: 1.06–1.21), with a marked upsurge relative to the slightly upward background trend before and after the fourth campaign (see Figure S2).

### 3.6 Cancer diagnoses in the National Cancer Registration Dataset

The number of bladder carcinoma in situ diagnosed according to the National Cancer Registration Dataset was 11% higher in the analysis period relative to the comparison period (RR 1.11, 95% CI: 1.05–1.17). However, there was little evidence of differences in the diagnosis of malignant bladder cancer (RR 0.97, 95% CI: 0.92–1.02), kidney and urinary tract cancer (RR 1.00, 95% CI: 0.95–1.05) or non‐invasive papillary carcinoma of the bladder (RR 1.00, 95% CI: 0.83–1.19). There were also no clear departures from the underlying trends, although this could have been masked by week to week variability (see Figure S3).

### 3.7 Early stage at diagnosis

There was a small, statistically non‐significant (*p* = 0.07) increase in the percentage of early‐stage bladder cancers in the analysis period, compared to the comparison period. 49.1% of cancers were early stage in the analysis period, compared to 46.3% in the comparison period (difference in percentage [analysis − comparison] 2.8%, 95% CI: −0.2–5.8). The percentage of early‐stage kidney and urinary tract cancers decreased slightly, from 54.4% in the comparison period to 53.9% in the analysis period (difference in percentage [analysis − comparison] ‐0.6%, 95% CI: −3.2%–2.1%). These findings appear consistent with changes in the trends over the period of the four campaigns (see Figure S4).

### 3.8 Diagnostics in secondary care

The number of ultrasounds, CT and MRI of the bladder and kidney was 10% higher in the analysis period, relative to the comparison period (RR 1.10, 95% CI: 1.06–1.14). There has been an upward trajectory in diagnostic activity in this area from 2012 to 2018 (see Figure S5).

### 3.9 Survival

The was some evidence of improved survival within 1 year for those diagnosed with bladder cancer in the analysis period, relative to the comparison period (HR 0.93, 95% CI: 0.83–0.99). Survival was similar for those diagnosed with kidney cancer in the two periods (HR 0.98, 95% CI: 0.91–1.05).

Sensitivity analyses did not demonstrate any significant differences between gender or age groups.

## 4 DISCUSSION

### 4.1 Key findings

This prospective evaluation of the fourth BCoC ‘BiP’ campaign showed an increase in bladder and kidney cancer symptom awareness amongst the population following the delivery of public health messages via a range of different media. There were also increases in GP attendances with haematuria and urgent suspected urological cancer referrals. Cancer diagnoses and changes in 1‐year survival were broadly in line with the longer term trends prior to and following the campaign. There was a small, statistically non‐significant increase in bladder cancer diagnoses from urgent cancer referrals, despite long‐term trends of falling incidence, and a non‐significant increase in the diagnoses of early‐stage bladder cancer. Underlying trends for cancer diagnoses, emergency presentations and diagnostic activity over the period of the fourth BiP campaigns did not seem to be significantly impacted by the campaigns.

### 4.2 Comparison with existing literature

The results of the evaluation of the fourth BCoC BiP campaign are consistent with the PHE report on the first three national campaigns (Kockelbergh, 2020). This report highlighted an increase in symptom recognition, GP attendances and urgent referrals for suspected urological cancer, with more mixed results for bladder and kidney cancers diagnosed and early‐stage diagnoses. There was evidence from this report of diminishing returns on the subsequent BCoC BiP campaigns relative to the early campaigns, which matches the findings of Lai et al. who assessed published evidences of evaluations for 11 BCoC campaigns across a range of tumour types (bowel, lung, bladder and kidney, breast and gastro‐oesophageal) (Lai et al., 2020).

The effectiveness of earlier BCoC BiP campaigns had been assessed by two small studies by Hughes‐Hallett et al. (2016) and Patel et al. (2019). Both evaluated the impacts of the first BCoC BiP campaigns on local referral patterns, diagnostic activity and cancer diagnoses, and both studies found an increase in referrals and diagnostic activity without a demonstrable increase in urological cancer diagnoses. Both studies were likely underpowered to identify any change in cancer diagnoses or survival as a result of earlier campaigns. They were also retrospective in nature, conducted at single NHS Trusts, using different analysis periods, and applied different methodologies to the national‐level evaluations of the campaigns. Consequently, they may not have been able to assess accurately the impact of a national campaign. However, national‐level analyses can mask local variation and important assertions were made by authors of both papers about the downstream impact of public awareness campaigns on cancer diagnostic services.

### 4.3 Strengths and limitations

This robust evaluation of the fourth BCoC BiP campaign assessed the impact of the public health messaging using a range of national level data sources, including PHE National Cancer Registration data and NHS England cancer data. The methodologies applied have been refined over a number of BCoC campaign evaluations following a similar model. The public health materials and messages have also been adapted and better targeted following the evaluation of previous BCoC BiP campaigns. The co‐occurrence of peaks of attendance with the campaign periods, which were each conducted at different times of year, strengthens the attribution of the change in patterns of presentation to the campaigns.

The use of pre‐existing, national‐level datasets for the evaluation of this campaign could also be considered a limitation, given that none was specifically collected for this study: Therefore, links to changes in cancer diagnoses are inferential rather than causal. Attribution bias could result from the assumption that changes in key measures between the analysis and comparison periods are assumed to be the result of the effect of the BCoC BiP campaign, when they occur for other reasons or are part of the long‐term trends seen in Figures 1, 2, 3. The changing incidence of bladder and kidney cancer in recent decades probably explain at least some of the effects of the BCoC BiP campaign, as well as factors unrelated to the campaigns such as the Fry and Turnbull effect on urological cancer diagnoses (Lovegrove et al., 2019). Without longer term data and follow‐up, it is unclear whether the changes seen as a result of the campaign will be sustained or not. Trend lines suggest the campaign's effects are short term in nature, and previous evaluations suggest there is evidence of a return to pre‐campaign levels after a washout period. Finally, despite being a large evaluation, the size of the sample may have meant that small changes in some of the metrics that we examined may not have been detectable.

### 4.4 Implications for policy and practice

Raising cancer symptom awareness and reducing barriers for GP attendances with symptoms potentially linked to an undiagnosed cancer has been one area of focus aimed at addressing the UK's relatively lower cancer survival compared to other high‐income countries (Forbes et al., 2015) and thus achieving the NHS England aim of early‐stage cancer diagnosis for 75% of patients by 2028 (NHS, 2019). The BCoC BiP campaign appears to be effective in increasing symptom awareness for bladder and kidney cancer and subsequent GP presentations in this and previous evaluations (Kockelbergh, 2020), in line with the effect of BCoC campaigns on other cancer types (Lai et al., 2020). Increased urgent suspected cancer referrals have been shown to reduce overall mortality and increase the rates of early‐stage cancer diagnosis (Round et al., 2020), which would suggest that the BCoC campaigns' small effect on urgent referrals might have a positive effect. However, the downstream campaign impacts on cancer diagnosis rates, the proportions of patients with an early diagnosis of kidney or bladder cancer and 1‐year survival are not clearly demonstrated in this study.

There are some possible explanations for the lack of clear translation in the benefits of increasing awareness of serious cancer symptoms, such as haematuria, on cancer diagnoses and outcomes. It had been thought that reducing the interval prior to presentation with haematuria may not make a significant difference to early‐stage diagnosis, although a recent study by Koo et al. showed that only 21% (95% CI 17%–25%) of 487 patients in the 2014 English National Cancer Diagnosis Audit presenting with haematuria were subsequently diagnosed with Stage 4 cancer. Health system factors may reduce the impact of the BCoC BiP campaigns; suspected urological urgent cancer referral pathways within the NHS are under significant pressure to diagnose and treat patients in a timely manner (Richardson et al., 2020), so additional primary care referrals without diagnostic capacity to meet the increased demand may not result in significantly earlier diagnoses. Further unanswered questions that remains include the cost‐effectiveness of these campaigns and whether the benefits (near and longer term) are enough to offset the increased primary and secondary care activity that results from these public health activities.

### 4.5 CONCLUSIONS

The fourth BCoC BiP campaign appears to have achieved its main aim of raising awareness of the importance of the symptoms of bladder and kidney cancer amongst the public and encouraging them to present to their GP. Downstream data show an increase in GP attendances and urgent cancer referral activity in the short‐term. It is unclear whether these more direct campaign effects impact on bladder and kidney cancer diagnoses, including early‐stage cancer diagnoses or whether changes observed are the result of long‐term trends in cancer incidence and survival.

## CONFLICT OF INTEREST

The authors declare no conflicts of interest.

## AUTHOR CONTRIBUTIONS

SM, SB, CH and WH conceived the study idea and analysis plan. CB, VM, CG, LP and LEB collected and organised the data. SB led on the analysis with input from CB and VM. SM, SB, CH and WH undertook data interpretation. SM drafted the manuscript. All authors read and commented on the draft manuscript, and all authors approved the final submitted version.

## Supporting information


**Figure S1.** Trend line for percentage of cancers diagnosed as an emergency between October 2012 – December 2018 (Solid line = bladder; dashed line = Kidney and unspecified urinary organ)

**Figure S2.** Trend line for number of cancer diagnoses in the Cancer Waiting Times (CWT) database between October 2012 – December 2018 (Solid line = bladder; dashed line = kidney and urinary tract; dotted line = urological [including prostate])

**Figure S3.** Trend line for number of cancer diagnoses in the NCRAS cancer registry between October 2012 – December 2018

**Figure S4.** Trend line for percentage of early stage cancer diagnoses for malignant bladder and kidney and urinary tract between October 2012 – December 2018

**Figure S5.** Trend line for number of ultrasounds, MRIs and CTs of the kidney and urinary tract between October 2012 – December 2018

**Table S1.** Results of metrics – ages 50 years and over

**Table S2.** Results of metrics – males

**Table S3.** Results of metrics – females

**Data S1.** STROBE checklist

**Data S2.** Online symptom awareness questionnaire

## Data Availability

This manuscript outlines analyses of a combination of publically available data (https://www.england.nhs.uk/statistics/statistical‐work‐areas/cancer‐waiting‐times/), data held by public bodies (National Cancer Registry, Hospital Episode Statistics, NHS digital datasets) and commercially collected data (THIN database, Kantar surveys).
