# Supplementary figures and images for: A prospective evaluation of the fourth national Be Clear on Cancer ‘Blood in Pee’ campaign in England
Source: Eur J Cancer Care (Engl). 2022 May 15;31(5):e13606. doi: 10.1111/ecc.13606 (PMC9539495; doi:10.1111/ecc.13606)

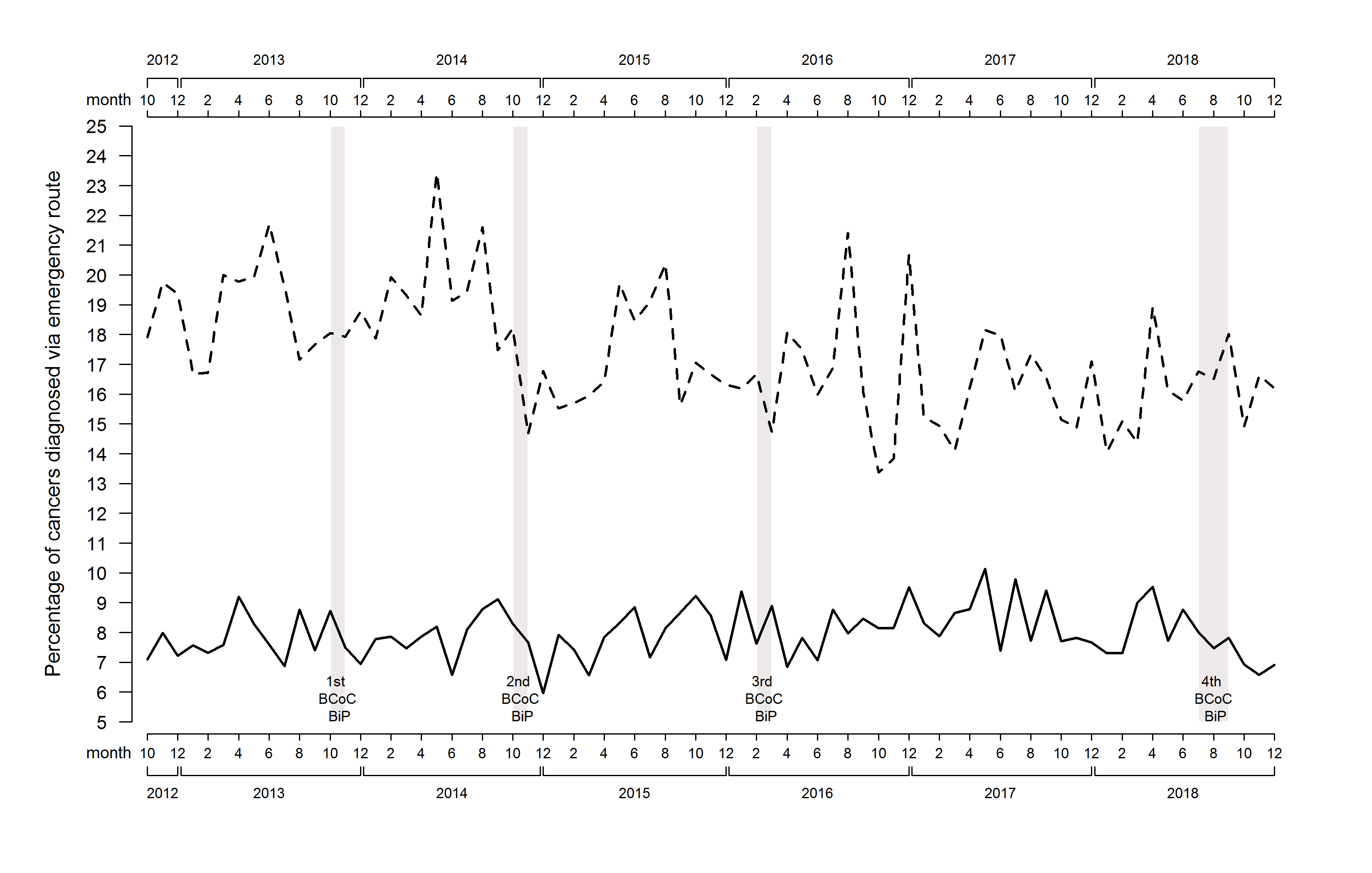

Supplement: Supplementary file 1 — Figure S1. Trend line for percentage of cancers diagnosed as an emergency between October 2012 – December 2018 (Solid line = bladder; dashed line = Kidney and unspecified urinary organ) [file ECC-31-e13606-s006.tiff]

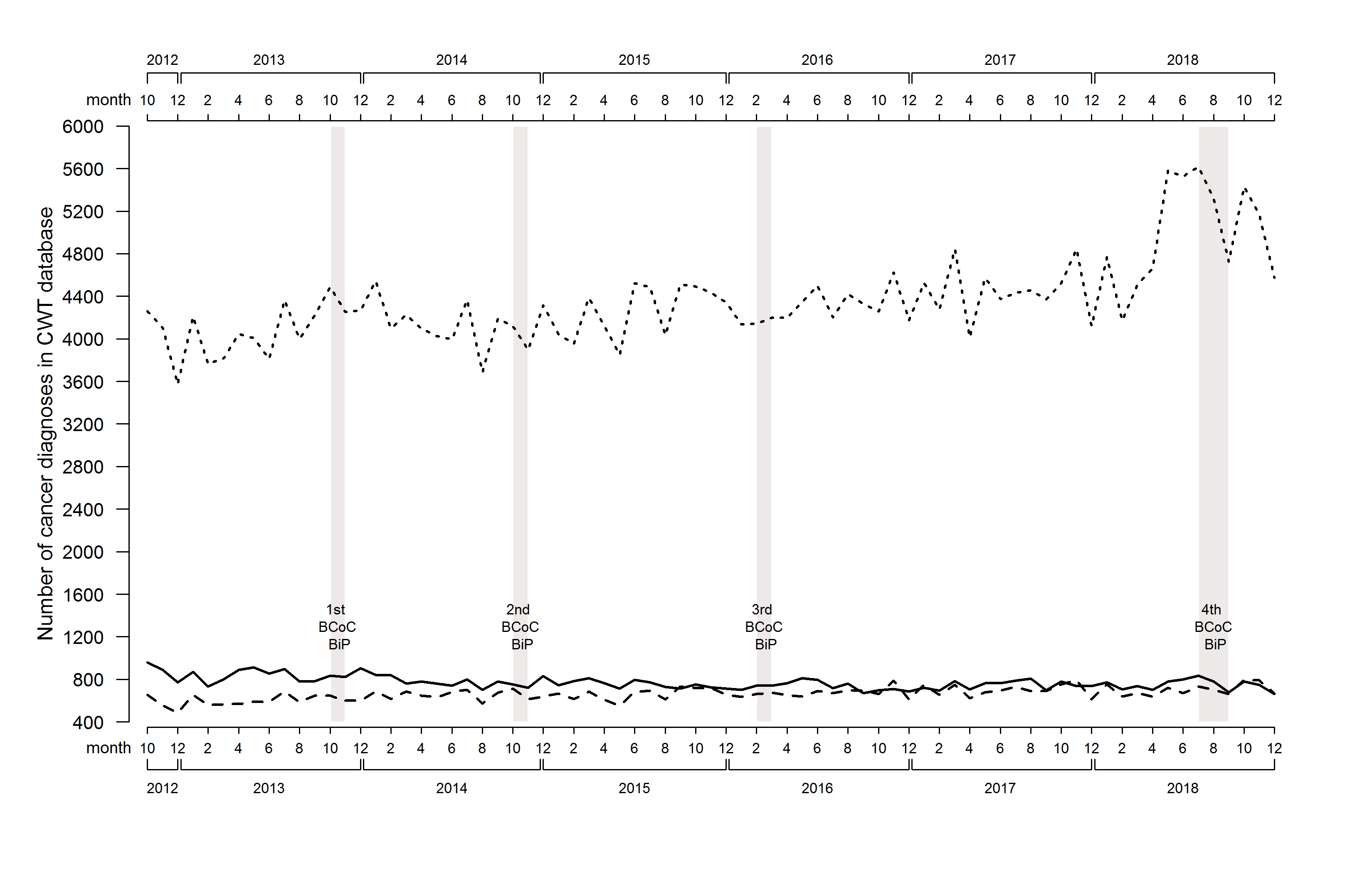

Supplement: Supplementary file 2 — Figure S2. Trend line for number of cancer diagnoses in the Cancer Waiting Times (CWT) database between October 2012 – December 2018 (Solid line = bladder; dashed line = kidney and urinary tract; dotted line = urological [including prostate]) [file ECC-31-e13606-s010.tiff]

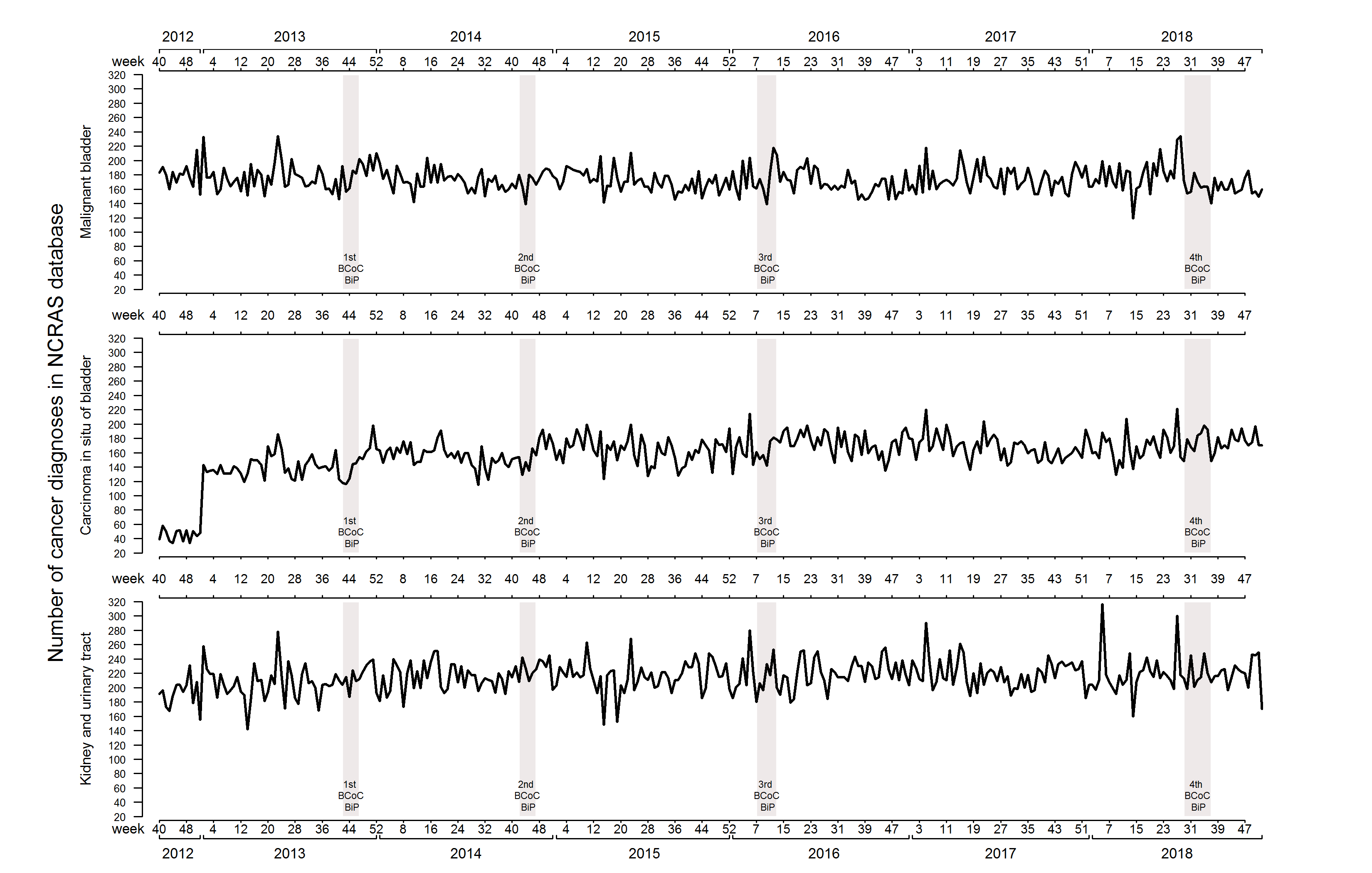

Supplement: Supplementary file 3 — Figure S3. Trend line for number of cancer diagnoses in the NCRAS cancer registry between October 2012 – December 2018 [file ECC-31-e13606-s003.tiff]

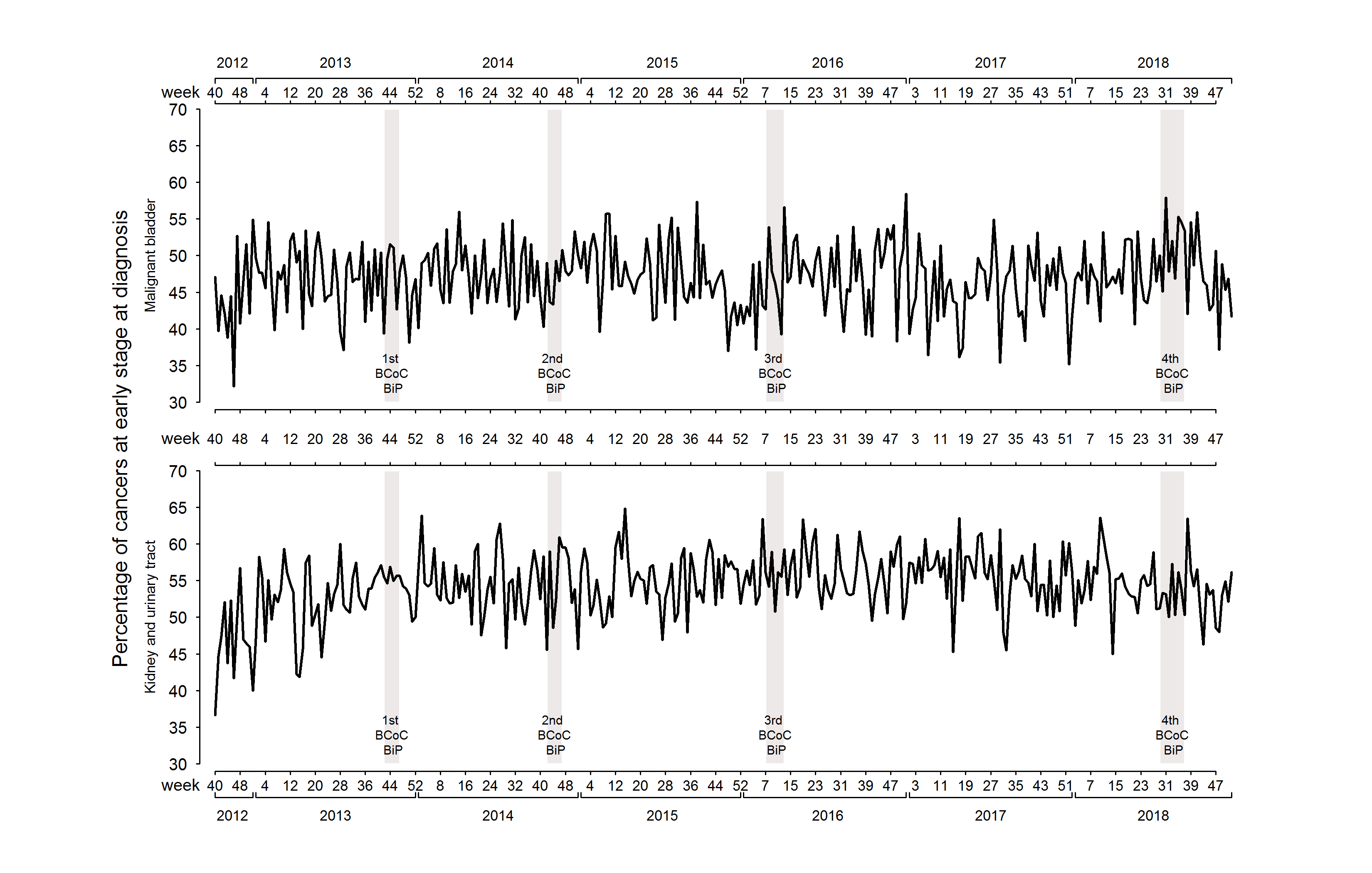

Supplement: Supplementary file 4 — Figure S4. Trend line for percentage of early stage cancer diagnoses for malignant bladder and kidney and urinary tract between October 2012 – December 2018 [file ECC-31-e13606-s001.tiff]

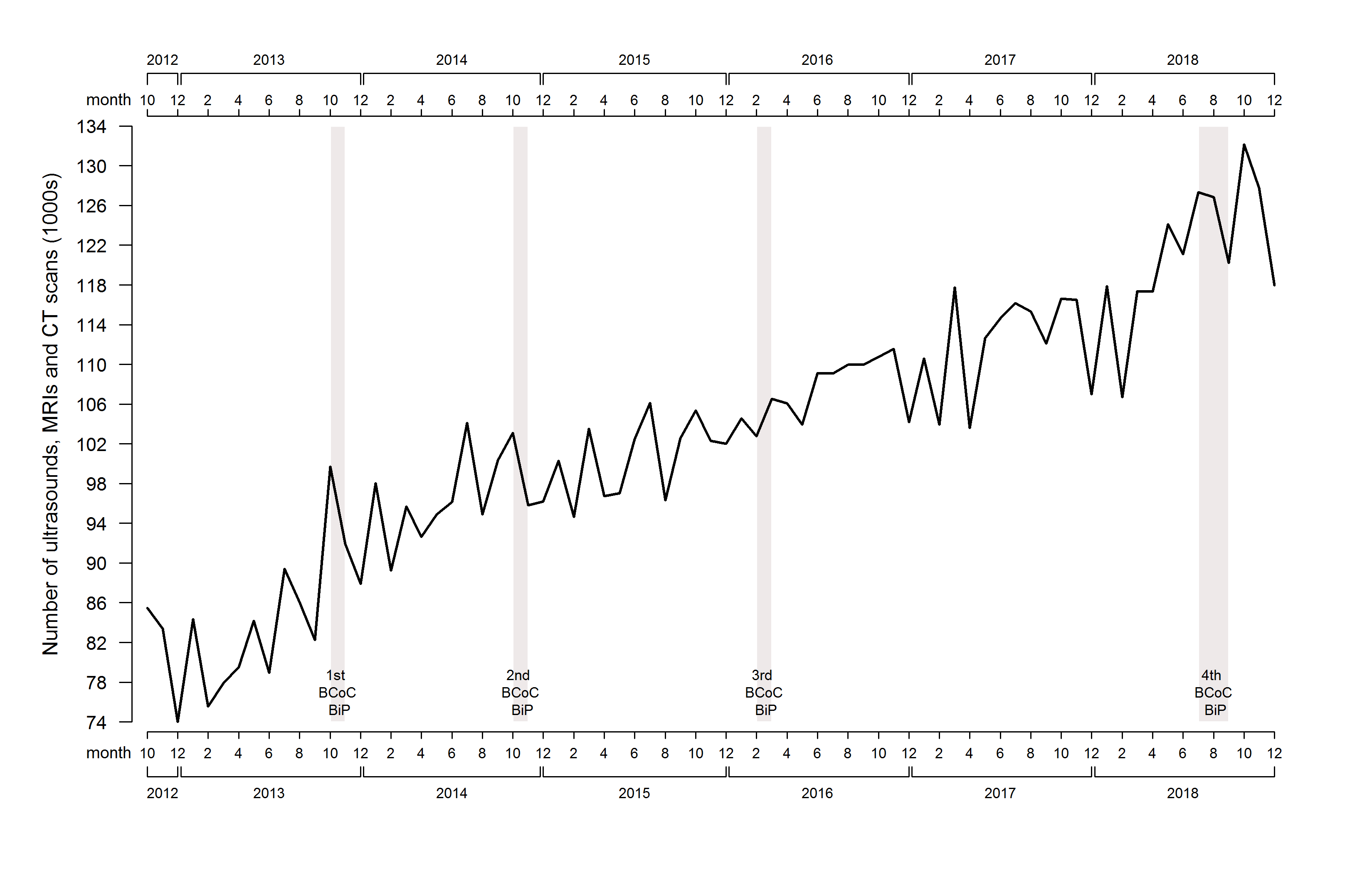

Supplement: Supplementary file 5 — Figure S5. Trend line for number of ultrasounds, MRIs and CTs of the kidney and urinary tract between October 2012 – December 2018 [file ECC-31-e13606-s004.tiff]
