# Supplementary material for: A prospective evaluation of the fourth national Be Clear on Cancer ‘Blood in Pee’ campaign in England
Source: Eur J Cancer Care (Engl). 2022 May 15;31(5):e13606. doi: 10.1111/ecc.13606 (PMC9539495; doi:10.1111/ecc.13606)
Supplement: Supplementary file 6 — Table S1. Results of metrics – ages 50 years and over [file ECC-31-e13606-s009.docx]

Supplementary table 1: results of metrics – ages 50 years and over

| **Metric** | **Type of symptom/referral/cancer** | **Comparison period** | **Analysis period** | **Statistic** | **Estimate (95% CI)** | **p value** |
| --- | --- | --- | --- | --- | --- | --- |
| GP attendances | Blood in pee | 0.44 attendances per practice per week | 0.50 attendances per practice per week | Rate ratio | 1.14 (1.02 to 1.26) | 0.02 |
| Urgent GP referrals | Suspected urological cancer | 57188 | 67718 | Rate ratio | 1.18 (1.09 to 1.29) | <0.001 |
| Cancer diagnosed from urgent GP referral for suspected urological cancer | Bladder | 1892 | 1904 | Rate ratio | 1.01 (0.91 to 1.11) | 0.90 |
|  | Kidney and urinary tract | 890 | 1010 | Rate ratio | 1.13 (0.99 to 1.30) | 0.08 |
|  | Urological cancer (including prostate) | 10410 | 11787 | Rate ratio | 1.13 (1.03 to 1.24) | 0.01 |
| Emergency cancer diagnoses | Bladder | 8.8% (466 of 5313) | 7.7% (415 of 5398) | Difference in percentage | -1.1% (-2.1% to -0.04%) | 0.04 |
|  | Kidney and unspecified urinary organ | 16.4% (375 of 2293) | 17.1% (408 of 2390) | Difference in percentage | 0.7% (-1.4% to 2.9%) | 0.51 |
| Cancer diagnoses in CWT database | Bladder | 2945 | 2902 | Rate ratio | 0.99 (0.91 to 1.07) | 0.72 |
|  | Kidney and urinary tract | 2631 | 2655 | Rate ratio | 1.01 (0.92 to 1.10) | 0.84 |
|  | Urological cancer (including prostate) | 17668 | 20114 | Rate ratio | 1.14 (1.07 to 1.21) | <0.001 |
| Cancer diagnoses in National Cancer Registration Dataset | Malignant bladder | 2487.75 | 2395.5 | Rate ratio | 0.96 (0.91 to 1.02) | 0.19 |
|  | Bladder carcinoma in situ | 2287.75 | 2545.75 | Rate ratio | 1.11 (1.05 to 1.18) | <0.001 |
|  | Kidney and urinary tract | 2955.5 | 2964.75 | Rate ratio | 1.00 (0.95 to 1.06) | 0.90 |
|  | pTa | 230 | 220.5 | Rate ratio | 0.96 (0.80 to 1.15) | 0.65 |
| Early stage at diagnosis | Malignant bladder | 46.4%  (1009.25 of 2175 staged cases) | 49.1%  (994.5 of 2024 staged cases) | Difference in percentage | 2.7% (-0.3% to 5.8%) | 0.08 |
|  | Kidney and urinary tract | 53.1%  (1310.5 of 2469 staged cases) | 52.7%  (1302.75 of 2473 staged cases) | Difference in percentage | -0.4% (-3.2% to 2.4%) | 0.78 |
| Diagnostics in secondary care | Ultrasounds, MRIs and CT scans | 320370 | 354060 | Rate ratio | 1.11 (1.06 to 1.15) | <0.001 |
| 1 year survival | Bladder | 70.4 | 70.4 | Hazard ratio | 0.93 (0.88 to 0.99) | 0.03 |
|  | Kidney | 77.5 | 78.4 | Hazard ratio | 0.99 (0.93 to 1.06) | 0.79 |
