# Supplementary material for: A prospective evaluation of the fourth national Be Clear on Cancer ‘Blood in Pee’ campaign in England
Source: Eur J Cancer Care (Engl). 2022 May 15;31(5):e13606. doi: 10.1111/ecc.13606 (PMC9539495; doi:10.1111/ecc.13606)
Supplement: Supplementary file 7 — Table S2. Results of metrics – males [file ECC-31-e13606-s002.docx]

Supplementary table 2: results of metrics – males

| **Metric** | **Type of symptom/referral/cancer** | **Comparison period** | **Analysis period** | **Statistic** | **Estimate (95% CI)** | **p value** |
| --- | --- | --- | --- | --- | --- | --- |
| GP attendances | Blood in pee | 0.37 attendances per practice per week | 0.46 attendances per practice per week | Rate ratio | 1.24 (1.11 to 1.40) | <0.001 |
| Urgent GP referrals | Suspected urological cancer | 48024 | 58167 | Rate ratio | 1.21 (1.11 to 1.32) | <0.001 |
| Cancer diagnosed from urgent GP referral for suspected urological cancer | Bladder | 1438 | 1492 | Rate ratio | 1.04 (0.91 to 1.18) | 0.57 |
|  | Kidney and urinary tract | 629 | 720 | Rate ratio | 1.14 (0.96 to 1.36) | 0.13 |
|  | Urological cancer (including prostate) | 9790 | 11162 | Rate ratio | 1.14 (1.04 to 1.25) | 0.01 |
| Emergency cancer diagnoses | Bladder | 7.7% (309 of 4017) | 6.4% (270 of 4214) | Difference in percentage | -1.3% (-2.4% to -0.2%) | 0.02 |
|  | Kidney and unspecified urinary organ | 16.2% (262 of 1615) | 15.5% (253 of 1636) | Difference in percentage | -0.8% (-3.3% to 1.8%) | 0.55 |
| Cancer diagnoses in CWT database | Bladder | 2203 | 2243 | Rate ratio | 1.02 (0.96 to 1.08) | 0.55 |
|  | Kidney and urinary tract | 1803 | 1887 | Rate ratio | 1.05 (0.94 to 1.16) | 0.39 |
|  | Urological cancer (including prostate) | 16254 | 18839 | Rate ratio | 1.16 (1.09 to 1.24) | <0.001 |
| Cancer diagnoses in National Cancer Registration Dataset | Malignant bladder | 1840.25 | 1811.25 | Rate ratio | 0.98 (0.92 to 1.05) | 0.63 |
|  | Bladder carcinoma in situ | 1796.5 | 2049 | Rate ratio | 1.14 (1.07 to 1.22) | <0.001 |
|  | Kidney and urinary tract | 2023.5 | 2068.25 | Rate ratio | 1.02 (0.96 to 1.09) | 0.48 |
|  | pTa | 177.25 | 176.75 | Rate ratio | 1.00 (0.81 to 1.23) | 0.98 |
| Early stage at diagnosis | Malignant bladder | 49.7%  (806.25 of 1623.75 staged cases) | 52.3%  (812.25 of 1553.75 staged cases) | Difference in percentage | 2.6% (-0.9% to 6.1%) | 0.14 |
|  | Kidney and urinary tract | 53.7%  (906 of 1687 staged cases) | 52.2%  (901.75 of 1728.75 staged cases) | Difference in percentage | -1.5% (-4.9% to 1.8%) | 0.37 |
| Diagnostics in secondary care | Ultrasounds, MRIs and CT scans | 226715 | 250365 | Rate ratio | 1.10 (1.07 to 1.14) | <0.001 |
| 1 year survival | Bladder | 74.2 | 75.7 | Hazard ratio | 0.93 (0.86 to 0.99) | 0.05 |
|  | Kidney | 79.4 | 79.6 | Hazard ratio | 0.95 (0.87 to 1.03) | 0.40 |
