# Supplementary material for: A prospective evaluation of the fourth national Be Clear on Cancer ‘Blood in Pee’ campaign in England
Source: Eur J Cancer Care (Engl). 2022 May 15;31(5):e13606. doi: 10.1111/ecc.13606 (PMC9539495; doi:10.1111/ecc.13606)
Supplement: Supplementary file 8 — Table S3. Results of metrics – females [file ECC-31-e13606-s007.docx]

Supplementary table 3: results of metrics – females

| **Metric** | **Type of symptom/referral/cancer** | **Comparison period** | **Analysis period** | **Statistic** | **Estimate (95% CI)** | **p value** |
| --- | --- | --- | --- | --- | --- | --- |
| GP attendances | Blood in pee | 0.22 attendances per practice per week | 0.23 attendances per practice per week | Rate ratio | 1.04 (0.84 to 1.29) | 0.70 |
| Urgent GP referrals | Suspected urological cancer | 15233 | 16255 | Rate ratio | 1.07 (0.97 to 1.17) | 0.17 |
| Cancer diagnosed from urgent GP referral for suspected urological cancer | Bladder | 496 | 458 | Rate ratio | 0.92 (0.81 to 1.05) | 0.22 |
|  | Kidney and urinary tract | 377 | 404 | Rate ratio | 1.07 (0.93 to 1.23) | 0.33 |
|  | Urological cancer (including prostate) | 873 | 862 | Rate ratio | 0.99 (0.90 to 1.08) | 0.79 |
| Emergency cancer diagnoses | Bladder | 11.2% (167 of 1493) | 11.1% (153 of 1384) | Difference in percentage | -0.1% (-2.4% to 2.2%) | 0.91 |
|  | Kidney and unspecified urinary organ | 16.3% (162 of 993) | 18.1% (188 of 1037) | Difference in percentage | 1.8% (-1.5% to 5.1%) | 0.28 |
| Cancer diagnoses in CWT database | Bladder | 816 | 742 | Rate ratio | 0.91 (0.82 to 1.00) | 0.06 |
|  | Kidney and urinary tract | 1123 | 1065 | Rate ratio | 0.95 (0.87 to 1.03) | 0.22 |
|  | Urological cancer (including prostate) | 1939 | 1807 | Rate ratio | 0.93 (0.84 to 1.03) | 0.18 |
| Cancer diagnoses in National Cancer Registration Dataset | Malignant bladder | 687.75 | 637.5 | Rate ratio | 0.93 (0.83 to 1.03) | 0.17 |
|  | Bladder carcinoma in situ | 593.25 | 604.5 | Rate ratio | 1.02 (0.91 to 1.14) | 0.75 |
|  | Kidney and urinary tract | 1247 | 1203.25 | Rate ratio | 0.96 (0.89 to 1.04) | 0.38 |
|  | pTa | 69.75 | 69.25 | Rate ratio | 0.99 (0.71 to 1.38) | 0.97 |
| Early stage at diagnosis | Malignant bladder | 36.8%  (215.25 of 584.5 staged cases) | 39.4%  (204.25 of 518.5 staged cases) | Difference in percentage | 2.6% (-3.2% to 8.3%) | 0.38 |
|  | Kidney and urinary tract | 55.6%  (576 of 1035.25 staged cases) | 56.8%  (570 of 1003.75 staged cases) | Difference in percentage | 1.2% (-3.2% to 5.5%) | 0.60 |
| Diagnostics in secondary care | Ultrasounds, MRIs and CT scans | 233235 | 256125 | Rate ratio | 1.10 (1.06 to 1.14) | <0.001 |
| 1 year survival | Bladder | 60.3 | 55.8 | Hazard ratio | 0.93 (0.84 to 1.04) | 0.28 |
|  | Kidney | 77.3 | 79.7 | Hazard ratio | 1.03 (0.92 to 1.15) | 0.51 |
