# Supplementary material for: A prospective evaluation of the fourth national Be Clear on Cancer ‘Blood in Pee’ campaign in England
Source: Eur J Cancer Care (Engl). 2022 May 15;31(5):e13606. doi: 10.1111/ecc.13606 (PMC9539495; doi:10.1111/ecc.13606)
Supplement: Supplementary file 10 — Data S2. Online symptom awareness questionnaire [file ECC-31-e13606-s008.docx]

CAWI

Questionnaire

Name of survey

**BCOC BiP 2018 Post wave**

Client name

**PHE**

Author(s)

**Denny, Michelle (TSMLP)**

**This questionnaire was written according to Kantar quality procedures**

checked by

| **Repeating study (if this survey has been previously conducted)** |  |
| --- | --- |
| **Name of survey** | **BCOC BiP 2018 Post wave** |
| **Language** | **English (United Kingdom)** |
| **Survey length (minutes)** | **15** |
| **Version** | **2** |
| **Author(s)** | **Denny, Michelle (TSMLP)** |
| **Contact** |  |
| **Panel** |  |
| **Sample size** | **Gross:**  **Net:** |
| **Sample description** |  |
| **Quota** |  |
| **If several countries: indicate the countries** |  |
| **If several targets** |  |
| **Check-in site** | [**http://www.kantar.com**](http://www.kantar.com/) |
| **Comments** |  |

# Index

**B001 - B001: INTRO / SCREENER**

Q001 - Intro:

Q002 - Sex:

Q003 - AGE_OPEN:

Q004 - REGION:

Q005 - SEG:

Q006 - TEXTBREAK:

**End B001 - B001: INTRO / SCREENER**

**B002 - B002: BLOOD IN PEE**

Q007 - CURRENT_CHECKS:

Q008 - CURRENT_ACTION_COLOUR:

Q009 - CURRENT_ACTION_BLOOD:

Q010 - HOW_MANY_TIMES:

Q016 - BiP_APPEARANCE:

Q014 - SYMPTOMS:

**End B002 - B002: BLOOD IN PEE**

**B003 - B003: PRE-STAGE CAMPAIGN MEASURES / ASCERTAINING NOISE**

Q011 - SEEN_ADS:

Q012 - SEEN_WHERE:

Q013 - DESCRIBE_ADS:

**End B003 - B003: PRE-STAGE CAMPAIGN MEASURES / ASCERTAINING NOISE**

**B004 - B004: KNOWLEDGE OF SIGNS AND SYMPTOMS OF KIDNEY/BLADDER CANCER**

Q015 - HOW_CONFIDENT:

Q017 - COMMITMENT_COMFORTABLE:

Q018 - COMMITMENT_IMPORTANT:

Q019 - COMMITMENT_ACTION:

Q020 - COMMITMENT_DIFFICULT:

Q021 - AGREE_WORRY:

Q022 - LIKELIHOOD_GP_ACTIONS:

Q023 - Q038: COMMITMENT_DIFFICULT_GP

Q024 - BiP_AWARENESS:

Q025 - Bladder_cancer_symptoms:

Q026 - AGREE_CANCER_STATEMENTS:

Q043 - GeneralCheck:

**End B004 - B004: KNOWLEDGE OF SIGNS AND SYMPTOMS OF KIDNEY/BLADDER CANCER**

**B005 - B005: CAM QUESTIONS**

Q027 - NOT_CAM:

Q028 - BCOC_LOGO:

**End B005 - B005: CAM QUESTIONS**

**B006 - B006: CAMPAIGN RECOGNITION**

Q038 - Campaign_Intro:

Q031 - SEEN_MALE_TV:

Q032 - SEEN_FEMALE_TV:

Q033 - SEEN_ANIMATION:

Q040 - SEEN_RADIO:

Q039 - SEEN_OOH:

Q042 - SEEN_LEAFLET:

Q034 - MAIN_MESSAGE:

Q035 - ADS_ATTS:

Q036 - ACTION_TAKEN:

Q037 - SEEN_BCOC:

**End B006 - B006: CAMPAIGN RECOGNITION**

**B007 - B007: ADDITIONAL DEMOGRAPHICS**

Q029 - ETHNICITY:

Q030 - MARITAL_STATUS:

**End B007 - B007: ADDITIONAL DEMOGRAPHICS**

| \| \| B001 - B001: INTRO / SCREENER \| Begin block \| \| --- \| --- \| \| \| --- \| --- \| --- \| |
| --- | --- | --- | --- |

| \| Q001 - Intro: \| Text \| \| --- \| --- \| |
| --- | --- | --- |
| \| Not back \| \| --- \| |
| \| Welcome to the survey.  This is a survey about health issues, carried out on behalf of Public Health England. It should take you about 10 minutes to complete. \| \| --- \| |

| \| Q002 - Sex: \| Single coded \| \| --- \| --- \| |
| --- | --- | --- |
| \| Not back \| \| --- \| |
| \| Are you? \| \| --- \| |
| \| Normal \| \| --- \| |
| \| 1 \| Male \| \| --- \| --- \| \| 2 \| Female \| \| 4 \| Other **Open *Position fixed* \| \| 3 \| I'd prefer not to say **Position fixed* \| |

| \| Q003 - AGE_OPEN: \| Numeric \| \| --- \| --- \| |
| --- | --- | --- |
| \| Not back \| Max = 90 \| \| --- \| |
| \| How old are you? \| \| --- \| |
| \| Screen in 50-69 years - online survey  Representative quotas on:  - Age  - SEG  - Gender  - Regional spread  Screen in 70+ years - F2F omnibus  Representative quotas on:  - Age  - SEG  - Gender  - Regional spread \| \| --- \| |
| \|  \| \| --- \| |

| \| Q004 - REGION: \| Single coded \| \| --- \| --- \| |
| --- | --- | --- |
| \| Not back \| \| --- \| |
| \| Whereabouts in the country do you live? \| \| --- \| |
| \| Normal \| \| --- \| |
| \| 1 \| North East \| \| --- \| --- \| \| 2 \| North West \| \| 3 \| Yorkshire and the Humber \| \| 4 \| East Midlands \| \| 5 \| West Midlands \| \| 6 \| East of England \| \| 7 \| South East excluding London \| \| 8 \| London \| \| 9 \| South West \| \| 10 \| Prefer not to say **Position fixed* \| |

| \| Q005 - SEG: \| Single coded \| \| --- \| --- \| |
| --- | --- | --- |
| \| Not back \| \| --- \| |
| \| Which of the following groups does the Chief Income Earner in your household belong to... \| \| --- \| |
| \| Scripter notes: Scripter notes: Text: “Who is the Chief Income Earner?”  Show hover over: “The person in the household with the largest income is the Chief Income Earner, however this income is obtained.”  For responses, SHOW TEXT IN BRACKETS WHEN CODE IS HOVERED OVER  1. Semi or unskilled manual worker (e.g. manual jobs that require no special training or qualifications; manual workers, apprentices to be skilled trades, caretaker, cleaner, nursery school assistant, park keeper, non-HGV driver, shop assistant etc.)  2. Skilled manual worker (e.g. Skilled Bricklayer, Carpenter, Plumber, Painter, Bus/Ambulance Driver, HGV driver, Unqualified assistant teacher, AA patrolman, pub/bar worker, etc.)  3. Supervisory or clerical/ Junior managerial/ Professional/ administrator (e.g. Office worker, Student Doctor, Foreman with 25+ employees, sales person, Student Teachers etc.)  4. Intermediate managerial/ Professional/ Administrative (e.g. Newly qualified (under 3 years) doctor, Solicitor, Board director small organisation, middle manager in large organisation, principle officer in civil Service/local government etc.)  5. Higher managerial/ Professional/Administrative (e.g. Established doctor, Solicitor, Board Director in large organisation (200+ employees, top level civil servant/public service employee, headteacher, etc.)  7. Retired and living on state pension only (If the Chief Income Earner is retired and has an occupational pension, please select according to their previous occupation)  8. Unemployed (for over 6 months) or not working due to long term sickness (If the Chief Income Earner is not in paid employment and has been out of work for less than 6 months, please select according to previous occupation) \| \| --- \| |
| \| Normal \| \| --- \| |
| \| 1 \| Semi or unskilled manual worker \| \| --- \| --- \| \| 2 \| Skilled manual worker \| \| 3 \| Supervisory or clerical/ Junior managerial/ Professional/ administrator \| \| 4 \| Intermediate managerial/ Professional/ Administrative \| \| 5 \| Higher managerial/ Professional/Administrative \| \| 6 \| Student \| \| 7 \| Retired and living on state pension only \| \| 8 \| Unemployed (for over 6 months) or not working due to long term sickness \| \| 9 \| Housewife / Househusband / Homemaker \| \| 10 \| Prefer not to say \| |

| \| Q006 - TEXTBREAK: \| Text \| \| --- \| --- \| |
| --- | --- | --- |
| \| Not back \| \| --- \| |
| \| For this survey we are interested in hearing your thoughts and beliefs on personal health issues. Please answer the questions as honestly as you can.  Please be assured that all the answers will be treated as strictly confidential, will be used for research purposes and you will not be identified by your answers in any way. All data will be processed in adherence to the Market Research Society’s Code of Conduct and Data Protection Act 2018. \| \| --- \| |

| \| \| B001 - B001: INTRO / SCREENER \| End block \| \| --- \| --- \| \| \| --- \| --- \| --- \| |
| --- | --- | --- | --- |

| \| \| B002 - B002: BLOOD IN PEE \| Begin block \| \| --- \| --- \| \| \| --- \| --- \| --- \| |
| --- | --- | --- | --- |

| \| Q007 - CURRENT_CHECKS: \| Single coded \| \| --- \| --- \| |
| --- | --- | --- |
| \| Not back \| \| --- \| |
| \| How often, if at all, do you check the colour of your pee before flushing the loo? \| \| --- \| |
| \| Normal \| \| --- \| |
| \| 1 \| Always \| \| --- \| --- \| \| 2 \| Usually \| \| 3 \| Occasionally \| \| 4 \| Rarely \| \| 5 \| Never \| \| 6 \| Don't know \| |

| \| Q008 - CURRENT_ACTION_COLOUR: \| Multi coded \| \| --- \| --- \| |
| --- | --- | --- |
| \| Not back \| Min = 1 \| \| --- \| |
| \| What, if anything, would you be likely to do if your pee was a different colour than you had seen before? \| \| --- \| |
| \| Normal \| \| --- \| |
| \| 1 \| Nothing - I wouldn't think it was worth worrying about **Position fixed *Exclusive* \| \| --- \| --- \| \| 2 \| Nothing - I'd ignore it and hope it goes away **Position fixed *Exclusive* \| \| 3 \| Wait and see if it happens again and then take action \| \| 4 \| Wait and see if another symptom develops and then take action \| \| 5 \| Visit your GP \| \| 6 \| Speak to a pharmacist \| \| 7 \| Speak to a nurse at your GP surgery / medical centre \| \| 8 \| Call NHS 111 \| \| 9 \| Speak about it with a friend/family member \| \| 10 \| Seek information / advice online \| \| 11 \| Seek an over the counter remedy \| \| 12 \| Something else **Open *Position fixed* \| \| 13 \| Don't know **Position fixed *Exclusive* \| |

| \| Q009 - CURRENT_ACTION_BLOOD: \| Multi coded \| \| --- \| --- \| |
| --- | --- | --- |
| \| Not back \| Min = 1 \| \| --- \| |
| \| Sometimes different coloured pee can indicate blood in your pee...  What, if anything, would you be likely to do if you noticed blood in your pee just once? \| \| --- \| |
| \| Normal \| \| --- \| |
| \| 1 \| Nothing - I wouldn't think it was worth worrying about **Position fixed *Exclusive* \| \| --- \| --- \| \| 2 \| Nothing - I'd ignore it and hope it goes away **Position fixed *Exclusive* \| \| 3 \| Wait and see if it happens again and then take action \| \| 4 \| Wait and see if another symptom develops and then take action \| \| 5 \| Visit your GP \| \| 6 \| Speak to a pharmacist \| \| 7 \| Speak to a nurse at your GP surgery / medical centre \| \| 8 \| Call NHS 111 \| \| 9 \| Speak about it with a friend/family member \| \| 10 \| Seek information / advice online \| \| 11 \| Seek an over the counter remedy \| \| 12 \| Something else **Open *Position fixed* \| \| 13 \| Don't know **Position fixed *Exclusive* \| |

| \| Q010 - HOW_MANY_TIMES: \| Single coded \| \| --- \| --- \| |
| --- | --- | --- |
| \| Not back \| \| --- \| |
| \| After how many times of seeing blood in your pee, would you go and visit your GP? \| \| --- \| |
| \| Normal \| \| --- \| |
| \| 10 \| Once \| \| --- \| --- \| \| 11 \| Twice \| \| 12 \| Three times \| \| 13 \| Four times \| \| 14 \| Five times \| \| 15 \| Six to 10 times \| \| 16 \| More than 10 times \| \| 17 \| Wouldn't go at all \| \| 9 \| Don't know \| |

| \| Q016 - BiP_APPEARANCE: \| Multi coded \| \| --- \| --- \| |
| --- | --- | --- |
| \| Not back \| Min = 1 \| \| --- \| |
| \| What do you imagine blood in your pee looks like?  *Please select all that you think apply* \| \| --- \| |
| \| Normal \| \| --- \| |
| \| 1 \| Orangey \| \| --- \| --- \| \| 2 \| Light pink \| \| 3 \| Dark red \| \| 4 \| Bright red \| \| 5 \| Reddish - purple \| \| 6 \| Dark brown \| \| 7 \| Streaks of pink/red/purple within normal colour \| \| 8 \| Cloudy \| \| 9 \| Spots of pink/red/purple within normal colour \| \| 10 \| Clots of blood in your pee \| \| 11 \| Don't know **Position fixed *Exclusive* \| |

| \| Q014 - SYMPTOMS: \| Open \| \| --- \| --- \| |
| --- | --- | --- |
| \| Not back \| \| --- \| |
| \| If a person sees blood in their pee, what do you think it could be a symptom of? \| \| --- \| |
| \|  \| \| --- \| |
| \| 999 \| Don't know **Position fixed *Exclusive* \| \| --- \| --- \| |

| \| \| B002 - B002: BLOOD IN PEE \| End block \| \| --- \| --- \| \| \| --- \| --- \| --- \| |
| --- | --- | --- | --- |

| \| \| B003 - B003: PRE-STAGE CAMPAIGN MEASURES / ASCERTAINING NOISE \| Begin block \| \| --- \| --- \| \| \| --- \| --- \| --- \| |
| --- | --- | --- | --- |

| \| Q011 - SEEN_ADS: \| Single coded \| \| --- \| --- \| |
| --- | --- | --- |
| \| Not back \| \| --- \| |
| \| Have you seen, heard or read any adverts, publicity or other types of information in the last couple of months which talked about seeing blood in your pee? \| \| --- \| |
| \| Normal \| \| --- \| |
| \| 1 \| Yes \| \| --- \| --- \| \| 2 \| No \| \| 3 \| Don't know \| |

| \| Q012 - SEEN_WHERE: \| Multi coded \| \| --- \| --- \| |
| --- | --- | --- |
| \| Not back \| Min = 1 \| \| --- \| |
| \| Where did you see or hear something about the subject of seeing blood in your pee?  Please select all that apply. \| \| --- \| |
| \| Ask only if **Q011 - SEEN_ADS**,1 \| \| --- \| |
| \| Normal \| \| --- \| |
| \| 1 \| TV programme or news \| \| --- \| --- \| \| 2 \| TV advertising \| \| 3 \| On catch up TV such as ITV Hub or All 4 \| \| 4 \| Radio programme or news \| \| 5 \| Radio advertising \| \| 6 \| National newspaper article \| \| 7 \| National newspaper advertising \| \| 8 \| Local newspaper article \| \| 9 \| Local newspaper advertising \| \| 10 \| Magazine article \| \| 11 \| Magazine advertising \| \| 12 \| On Facebook \| \| 13 \| Social networking sites other than Facebook \| \| 14 \| NetDoctor \| \| 15 \| NHS Choices website \| \| 16 \| Website other than NHS Choices \| \| 17 \| Advertising on the internet \| \| 18 \| In a GP surgery \| \| 19 \| In a hospital \| \| 20 \| In a pharmacy/chemist \| \| 21 \| TV screens in a GP surgery \| \| 22 \| On a bag from pharmacy / chemist \| \| 23 \| Poster/billboard/bus shelter \| \| 24 \| On buses/other transport \| \| 25 \| Something sent to me through the door \| \| 26 \| Word of mouth \| \| 27 \| Leisure centres / health clubs \| \| 28 \| Event in a shopping centre \| \| 29 \| In a washroom/toilet \| \| 30 \| In football programmes \| \| 31 \| Other (specify) **Open* \| \| 32 \| Don't know \| |

| \| Q013 - DESCRIBE_ADS: \| Open \| \| --- \| --- \| |
| --- | --- | --- |
| \| Not back \| \| --- \| |
| \| Can you describe the advertising or publicity you have seen recently on the subject of seeing blood in your pee, including everything it showed or told you? \| \| --- \| |
| \| ASK IF YES AT PREVIOUS QUESTION \| \| --- \| |
| \|  \| \| --- \| |
| \| 999 \| Don't know **Position fixed *Exclusive* \| \| --- \| --- \| |

| \| \| B003 - B003: PRE-STAGE CAMPAIGN MEASURES / ASCERTAINING NOISE \| End block \| \| --- \| --- \| \| \| --- \| --- \| --- \| |
| --- | --- | --- | --- |

| \| \| B004 - B004: KNOWLEDGE OF SIGNS AND SYMPTOMS OF KIDNEY/BLADDER CANCER \| Begin block \| \| --- \| --- \| \| \| --- \| --- \| --- \| |
| --- | --- | --- | --- |

| \| Q015 - HOW_CONFIDENT: \| Single coded \| \| --- \| --- \| |
| --- | --- | --- |
| \| Not back \| \| --- \| |
| \| How confident are you that you know what blood in pee could be a sign of? \| \| --- \| |
| \| SKIP IF SELECTED DON'T KNOW AT PREVIOUS \| \| --- \| |
| \| Normal \| \| --- \| |
| \| 1 \| Very confident \| \| --- \| --- \| \| 2 \| Fairly confident \| \| 3 \| Not very confident \| \| 4 \| Not at all confident, I have just guessed \| \| 5 \| Don't know \| |

| \| Q017 - COMMITMENT_COMFORTABLE: \| Single coded \| \| --- \| --- \| |
| --- | --- | --- |
| \| Not back \| \| --- \| |
| \| It is suggested to check the colour of your pee every time you go to the toilet.  If you checked the colour of your pee every time you went to the toilet, do you think that would make you feel…? \| \| --- \| |
| \| Normal \| \| --- \| |
| \| 1 \| 0 - I would feel very uncomfortable with this \| \| --- \| --- \| \| 2 \| 1 \| \| 3 \| 2 \| \| 4 \| 3 \| \| 5 \| 4 \| \| 6 \| 5 \| \| 7 \| 6 \| \| 8 \| 7 \| \| 9 \| 8 \| \| 10 \| 9 \| \| 11 \| 10 - I would feel very comfortable with this \| |

| \| Q018 - COMMITMENT_IMPORTANT: \| Single coded \| \| --- \| --- \| |
| --- | --- | --- |
| \| Not back \| \| --- \| |
| \| In the context of all your priorities in life, where do you place the importance of checking the colour of your pee before you flush? \| \| --- \| |
| \| Normal \| \| --- \| |
| \| 1 \| 0 - It is not at all important \| \| --- \| --- \| \| 2 \| 1 \| \| 3 \| 2 \| \| 4 \| 3 \| \| 5 \| 4 \| \| 6 \| 5 \| \| 7 \| 6 \| \| 8 \| 7 \| \| 9 \| 8 \| \| 10 \| 9 \| \| 11 \| 10 - It is very important \| |

| \| Q019 - COMMITMENT_ACTION: \| Single coded \| \| --- \| --- \| |
| --- | --- | --- |
| \| Not back \| \| --- \| |
| \| When thinking about checking the colour of your pee every time you flush, would you say... \| \| --- \| |
| \| Normal \| \| --- \| |
| \| 1 \| 0 - I definitely wouldn't do this \| \| --- \| --- \| \| 2 \| 1 \| \| 3 \| 2 \| \| 4 \| 3 \| \| 5 \| 4 \| \| 6 \| 5 - There are reasons I would, but also reasons I wouldn't \| \| 7 \| 6 \| \| 8 \| 7 \| \| 9 \| 8 \| \| 10 \| 9 \| \| 11 \| 10 - I definitely would do this \| |

| \| Q020 - COMMITMENT_DIFFICULT: \| Single coded \| \| --- \| --- \| |
| --- | --- | --- |
| \| Not back \| \| --- \| |
| \| To what extent do you agree or disagree that it would be difficult to check the colour of your pee every time you flush? \| \| --- \| |
| \| Normal \| \| --- \| |
| \| 1 \| 0 - Strongly disagree \| \| --- \| --- \| \| 2 \| 1 \| \| 3 \| 2 \| \| 4 \| 3 \| \| 5 \| 4 \| \| 6 \| 5 \| \| 7 \| 6 \| \| 8 \| 7 \| \| 9 \| 8 \| \| 10 \| 9 \| \| 11 \| 10 - Strongly agree \| |

| \| Q021 - AGREE_WORRY: \| Matrix \| \| --- \| --- \| |
| --- | --- | --- |
| \| Not back \| Number of rows: 3 \| Number of columns: 5 \| \| --- \| |
| \| Below are some statements that are sometimes made about seeing blood in your pee. How much do you agree or disagree with each of them? \| \| --- \| |
| \| Dynamic grid \| \| --- \| |
| \| Rows: Normal \| Columns: Normal \| \| --- \| |
| \| Rendered as Dynamic Grid \| \| --- \| |
| \|  \| Strongly disagree \| Disagree \| Agree \| Strongly agree \| Don't know \| \| --- \| --- \| --- \| --- \| --- \| --- \| \| Blood in pee could be a sign of cancer \| 🔾 \| 🔾 \| 🔾 \| 🔾 \| 🔾 \| \| I wouldn't worry about having blood in my pee if I had no other symptoms \| 🔾 \| 🔾 \| 🔾 \| 🔾 \| 🔾 \| \| I would only do something about blood in my pee if I saw it several times \| 🔾 \| 🔾 \| 🔾 \| 🔾 \| 🔾 \| |

| \| Q022 - LIKELIHOOD_GP_ACTIONS: \| Matrix \| \| --- \| --- \| |
| --- | --- | --- |
| \| Not back \| Number of rows: 14 \| Number of columns: 4 \| \| --- \| |
| \| Sometimes people put off going to see the GP, even when they have a symptom that they think might  be serious.  How much, if at all, do the following reasons apply to you? \| \| --- \| |
| \| Rows: Normal \| Columns: Normal \| \| --- \| |
| \| Rendered as Dynamic Grid \| \| --- \| |
| \|  \| A lot \| A little \| Not at all \| Don't know  **Position fixed* \| \| --- \| --- \| --- \| --- \| --- \| \| I don’t want to be thought of as a hypochondriac \| 🔾 \| 🔾 \| 🔾 \| 🔾 \| \| I would feel embarrassed \| 🔾 \| 🔾 \| 🔾 \| 🔾 \| \| I would be worried my GP will think I am wasting their time \| 🔾 \| 🔾 \| 🔾 \| 🔾 \| \| I would prefer not to know what it is \| 🔾 \| 🔾 \| 🔾 \| 🔾 \| \| It’s too difficult to get an appointment \| 🔾 \| 🔾 \| 🔾 \| 🔾 \| \| I’m too busy / can’t find the time to get to the doctors \| 🔾 \| 🔾 \| 🔾 \| 🔾 \| \| I would only go if I was sure it was something serious \| 🔾 \| 🔾 \| 🔾 \| 🔾 \| \| I would go online to get information and advice before I would consider going to see a GP \| 🔾 \| 🔾 \| 🔾 \| 🔾 \| \| I would talk to friends or family to get information and advice before I would consider going to see a GP \| 🔾 \| 🔾 \| 🔾 \| 🔾 \| \| I don’t want to be lectured by the GP about my lifestyle \| 🔾 \| 🔾 \| 🔾 \| 🔾 \| \| I would be scared to find out that it is something serious \| 🔾 \| 🔾 \| 🔾 \| 🔾 \| \| I would be scared to find out that I have an illness that might not be treatable \| 🔾 \| 🔾 \| 🔾 \| 🔾 \| \| I would worry about the impact of bad news on my friends and family \| 🔾 \| 🔾 \| 🔾 \| 🔾 \| \| I would rather not know if I have a serious illness like cancer \| 🔾 \| 🔾 \| 🔾 \| 🔾 \| |

| \| Q023 - Q038: COMMITMENT_DIFFICULT_GP \| Single coded \| \| --- \| --- \| |
| --- | --- | --- |
| \| Not back \| \| --- \| |
| \| When thinking about visiting a GP as soon as possible the first time you saw blood in your pee, would you say... \| \| --- \| |
| \| Normal \| \| --- \| |
| \| 1 \| 0 - I definitely wouldn't do this \| \| --- \| --- \| \| 2 \| 1 \| \| 3 \| 2 \| \| 4 \| 3 \| \| 5 \| 4 \| \| 6 \| 5 \| \| 7 \| 6 \| \| 8 \| 7 \| \| 9 \| 8 \| \| 10 \| 9 \| \| 11 \| 10 - I definitely would do this \| |

| \| Q024 - BiP_AWARENESS: \| Single coded \| \| --- \| --- \| |
| --- | --- | --- |
| \| Not back \| \| --- \| |
| \| Having blood in your pee is one of the symptoms of kidney and bladder cancers...  Were you aware of this before today? \| \| --- \| |
| \| Normal \| \| --- \| |
| \| 1 \| Yes \| \| --- \| --- \| \| 2 \| No \| \| 3 \| Don't know \| |

| \| Q025 - Bladder_cancer_symptoms: \| Open \| \| --- \| --- \| |
| --- | --- | --- |
| \| Not back \| \| --- \| |
| \| Aside from having blood in your pee, what other symptoms of kidney and bladder cancers are you aware of? \| \| --- \| |
| \|  \| \| --- \| |

| \| Q026 - AGREE_CANCER_STATEMENTS: \| Matrix \| \| --- \| --- \| |
| --- | --- | --- |
| \| Not back \| Number of rows: 7 \| Number of columns: 5 \| \| --- \| |
| \| Below are some statements that are sometimes made about kidney or bladder cancer, how much do you agree or disagree with each statement? \| \| --- \| |
| \| Rows: Normal \| Columns: Normal \| \| --- \| |
| \| Rendered as Dynamic Grid \| \| --- \| |
| \|  \| Strongly disagree \| Disagree \| Agree \| Strongly agree \| Don't know \| \| --- \| --- \| --- \| --- \| --- \| --- \| \| If kidney or bladder cancer is diagnosed early it is more likely to be treatable \| 🔾 \| 🔾 \| 🔾 \| 🔾 \| 🔾 \| \| Going to my GP early with a symptom of kidney or bladder cancer makes no difference to my chances of surviving cancer \| 🔾 \| 🔾 \| 🔾 \| 🔾 \| 🔾 \| \| Going to my GP early with a symptom of kidney or bladder cancer provides reassurance that the issue is now being addressed \| 🔾 \| 🔾 \| 🔾 \| 🔾 \| 🔾 \| \| Most cancer treatment is terrible, even worse than death \| 🔾 \| 🔾 \| 🔾 \| 🔾 \| 🔾 \| \| Women are more likely to develop bladder cancer than men \| 🔾 \| 🔾 \| 🔾 \| 🔾 \| 🔾 \| \| Women are more likely to develop kidney cancer than men \| 🔾 \| 🔾 \| 🔾 \| 🔾 \| 🔾 \| \| I would not want to know if I have kidney or bladder cancer \| 🔾 \| 🔾 \| 🔾 \| 🔾 \| 🔾 \| |

| \| Q043 - GeneralCheck: \| Single coded \| \| --- \| --- \| |
| --- | --- | --- |
| \| Not back \| \| --- \| |
| \| How much do you agree or disagree with the following statement?  I make a point of checking my body for changes that could be a sign of cancer. \| \| --- \| |
| \| Normal \| \| --- \| |
| \| 1 \| Strongly agree \| \| --- \| --- \| \| 2 \| Agree \| \| 3 \| Disagree \| \| 4 \| Strongly disagree \| \| 5 \| Don't know **Position fixed* \| |
| \| **Scripter notes:** Flip scale for half sample except DK \| \| --- \| |

| \| \| B004 - B004: KNOWLEDGE OF SIGNS AND SYMPTOMS OF KIDNEY/BLADDER CANCER \| End block \| \| --- \| --- \| \| \| --- \| --- \| --- \| |
| --- | --- | --- | --- |

| \| \| B005 - B005: CAM QUESTIONS \| Begin block \| \| --- \| --- \| \| \| --- \| --- \| --- \| |
| --- | --- | --- | --- |

| \| Q027 - NOT_CAM: \| Multi coded \| \| --- \| --- \| |
| --- | --- | --- |
| \| Not back \| Min = 1 \| \| --- \| |
| \| Have you, your family or close friends had bladder or kidney cancer? \| \| --- \| |
| \| Normal \| \| --- \| |
| \| 1 \| You \| \| --- \| --- \| \| 2 \| Partner \| \| 3 \| Close family member \| \| 4 \| Other family member \| \| 5 \| Close friend \| \| 6 \| Other friend \| \| 7 \| None **Position fixed *Exclusive* \| \| 8 \| Don't know **Position fixed *Exclusive* \| \| 9 \| Prefer not to say **Position fixed *Exclusive* \| |

| \| Q028 - BCOC_LOGO: \| Single coded \| \| --- \| --- \| |
| --- | --- | --- |
| \| Not back \| \| --- \| |
| \| Have you seen this logo before today? You may have seen it in a different colour to the one shown here. \| \| --- \| |
| \| SHOW ‘BE CLEAR ON CANCER’ LOGO IN  BLACK AND WHITE \| \| --- \| |
| \| Normal \| \| --- \| |
| \| 1 \| Yes \| \| --- \| --- \| \| 2 \| No \| \| 3 \| Don't know \| |

| \| \| B005 - B005: CAM QUESTIONS \| End block \| \| --- \| --- \| \| \| --- \| --- \| --- \| |
| --- | --- | --- | --- |

| \| \| B006 - B006: CAMPAIGN RECOGNITION \| Begin block \| \| --- \| --- \| \| \| --- \| --- \| --- \| |
| --- | --- | --- | --- |

| \| Q038 - Campaign_Intro: \| Text \| \| --- \| --- \| |
| --- | --- | --- |
| \| Not back \| \| --- \| |
| \| Now you will be shown some ads. Please watch carefully as we will be asking some questions on your views on the adverts, whether you have seen them before or not. \| \| --- \| |

| \| Q031 - SEEN_MALE_TV: \| Multi coded \| \| --- \| --- \| |
| --- | --- | --- |
| \| Not back \| Min = 1 \| \| --- \| |
| \| Have you seen this ad in the last couple of months?  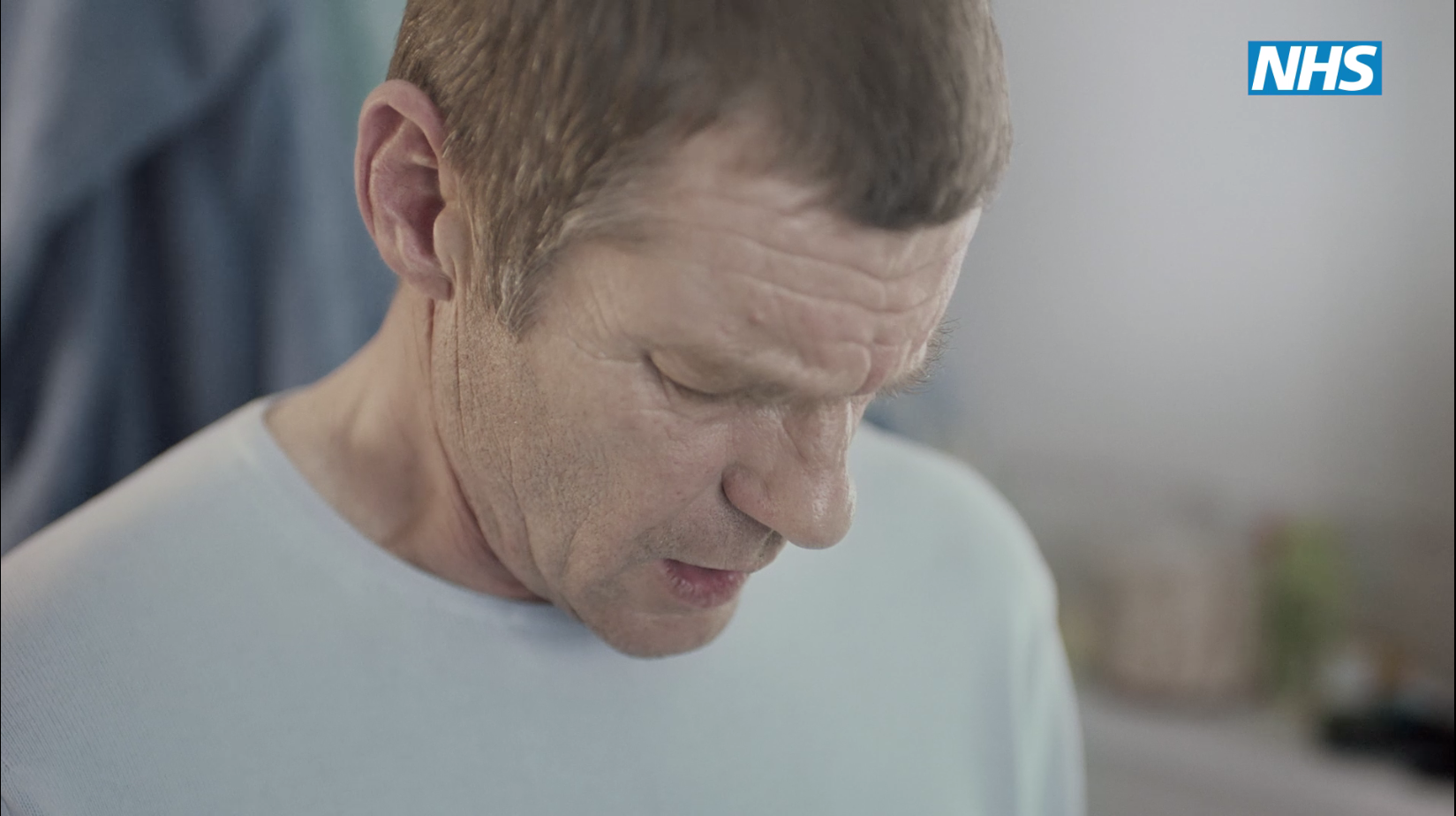 \| \| --- \| |
| \| SHOW MALE 30s 'BLOOD IN PEE' TV AD \| \| --- \| |
| \| Normal \| \| --- \| |
| \| 1 \| Yes, on TV \| \| --- \| --- \| \| 2 \| Yes, on catch up TV (e.g. ITV Hub, All 4) \| \| 3 \| Yes, online (e.g. YouTube) \| \| 4 \| Yes, on social media (e.g. Facebook, Twitter) \| \| 5 \| Yes, but I'm not sure where **Position fixed *Exclusive* \| \| 6 \| No **Position fixed *Exclusive* \| \| 999 \| Don't know **Position fixed *Exclusive* \| |

| \| Q032 - SEEN_FEMALE_TV: \| Multi coded \| \| --- \| --- \| |
| --- | --- | --- |
| \| Not back \| Min = 1 \| \| --- \| |
| \| Have you seen this ad in the last couple of months?  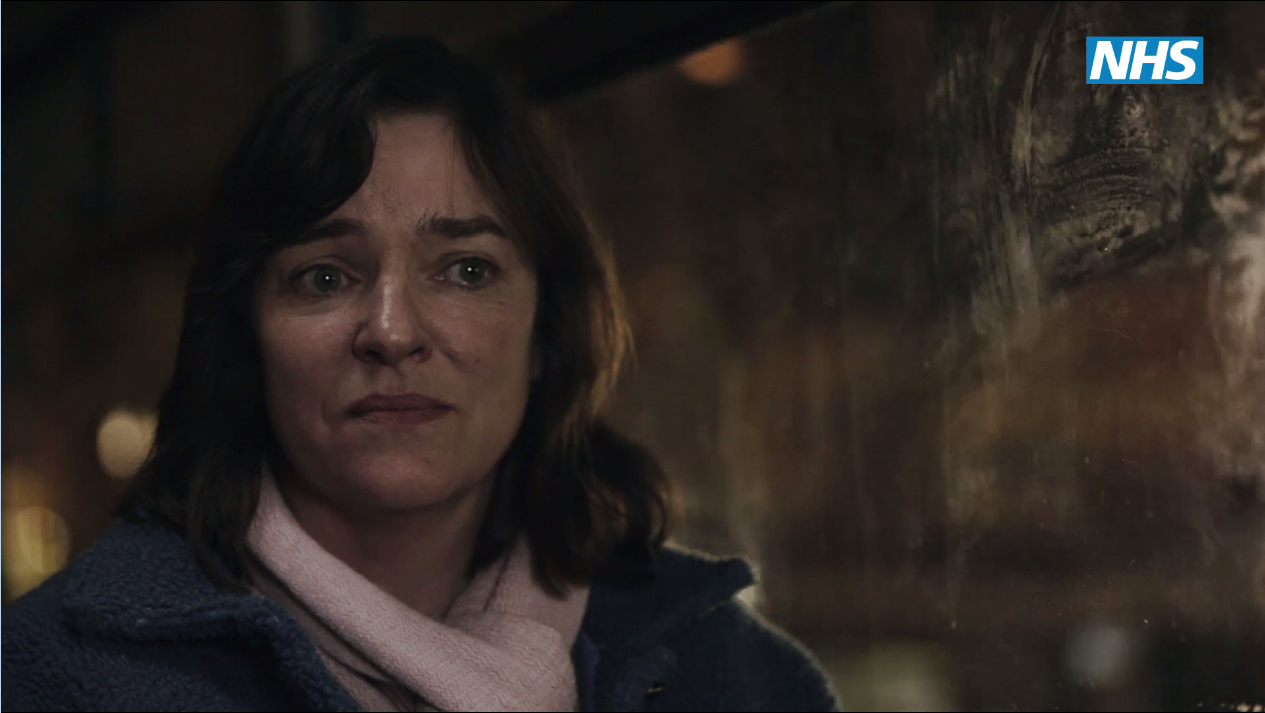 \| \| --- \| |
| \| SHOW FEMALE 30s 'BLOOD IN PEE' TV AD \| \| --- \| |
| \| Normal \| \| --- \| |
| \| 1 \| Yes, on TV \| \| --- \| --- \| \| 2 \| Yes, on catch up TV (e.g. ITV Hub, All 4) \| \| 3 \| Yes, online (e.g. YouTube) \| \| 4 \| Yes, on social media (e.g. Facebook, Twitter) \| \| 5 \| Yes, but I'm not sure where **Position fixed *Exclusive* \| \| 6 \| No **Position fixed *Exclusive* \| \| 999 \| Don't know **Position fixed *Exclusive* \| |

| \| Q033A - SEEN_ANIMATION: \| Single coded \| \| --- \| --- \| |
| --- | --- | --- |
| \| Not back \| Min = 1 \| \| --- \| |
| \| Have you seen these animations or videos online in the last couple of months?  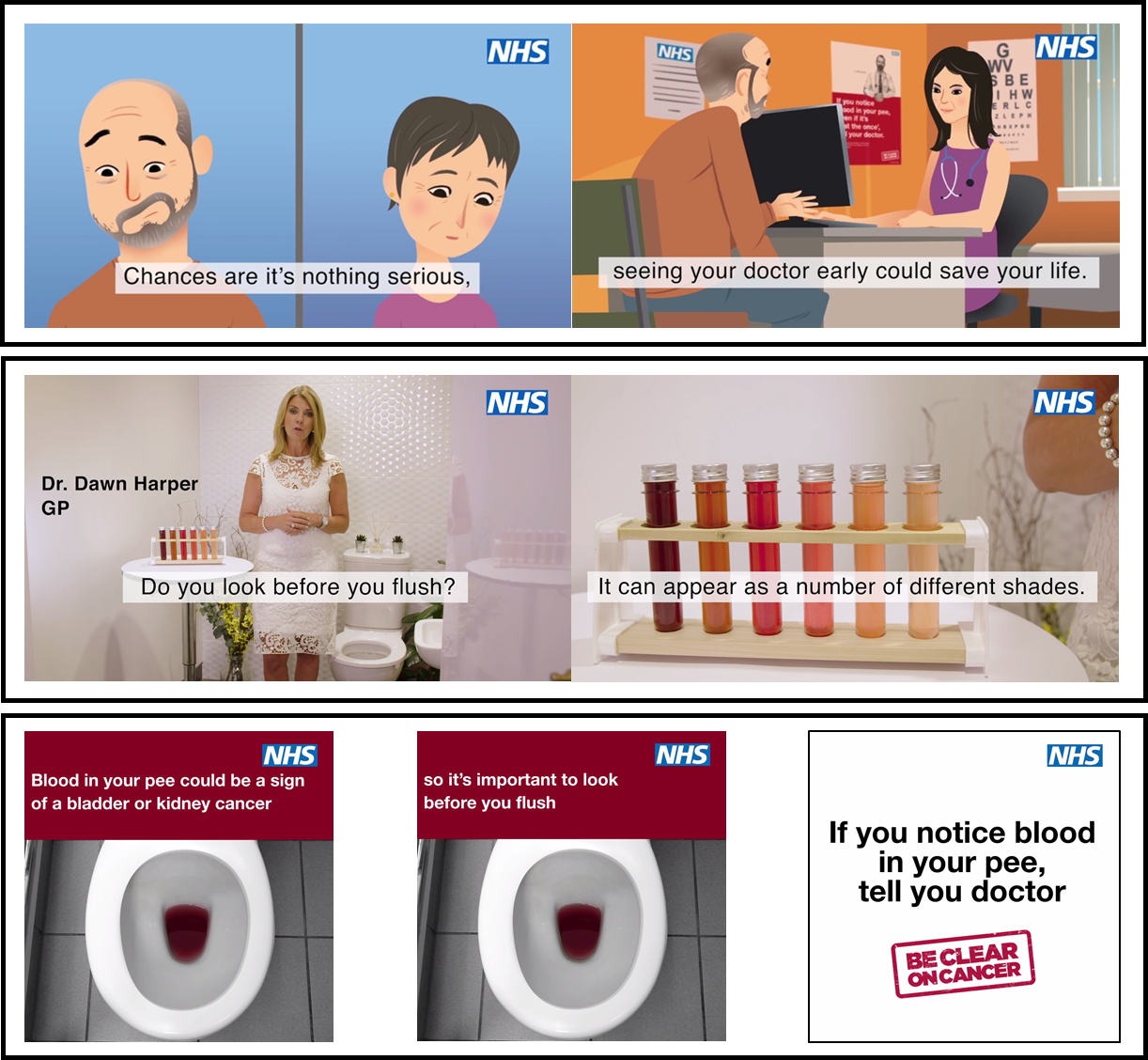 \| \| --- \| |
| \| SHOW AS GRID WITH YES / NO RESPONSE NEXT TO EACH \| \| --- \| |
| \| Normal \| \| --- \| |
| \| 1 \| Yes \| \| --- \| --- \| \| 2 \| No \| \| 999 \| Don't know **Position fixed* \| |

| \| Q033B - SEEN_ANIMATION: \| Multi coded \| \| --- \| --- \| |
| --- | --- | --- |
| \| Not back \| Min = 1 \| \| --- \| |
| \| Where have you seen the online animations and videos?  Please select all that apply \| \| --- \| |
| \| SHOW THUMBNAIL OF ADS SELECTED AS CODE 1 YES AT PREVIOUS  SKIP IF SELECT CODE 2 NO FOR ALL 3 ADS AT PREVIOUS \| \| --- \| |
| \| Normal \| \| --- \| |
| \| 1 \| On YouTube \| \| --- \| --- \| \| 2 \| On Facebook \| \| 3 \| On Twitter \| \| 4 \| Somewhere else online \| \| 5 \| Somewhere else not online \| \| 6 \| Not sure where \| |
| \| Q040 - SEEN_RADIO: \| Single coded \| \| --- \| --- \| |
| \| Not back \| \| --- \| |
| \| Have you heard this ad on the radio, or anything similar, in the last couple of months? \| \| --- \| |
| \| PLAY 1 RADIO AD PER RESPONDENT, RANDOMISE ORDER  RADIO 1  RADIO 2 \| \| --- \| |
| \| Normal \| \| --- \| |
| \| 1 \| Yes, heard this ad \| \| --- \| --- \| \| 2 \| Yes, heard something similar to this ad \| \| 3 \| No \| \| 999 \| Don't know \| |

| \| Q039 - SEEN_OOH: \| Multi coded \| \| --- \| --- \| |
| --- | --- | --- |
| \| Not back \| Min = 1 \| \| --- \| |
| \| Have you seen any of these ads, or something similar, in the last couple of months? If yes, where do you recall seeing these ads?  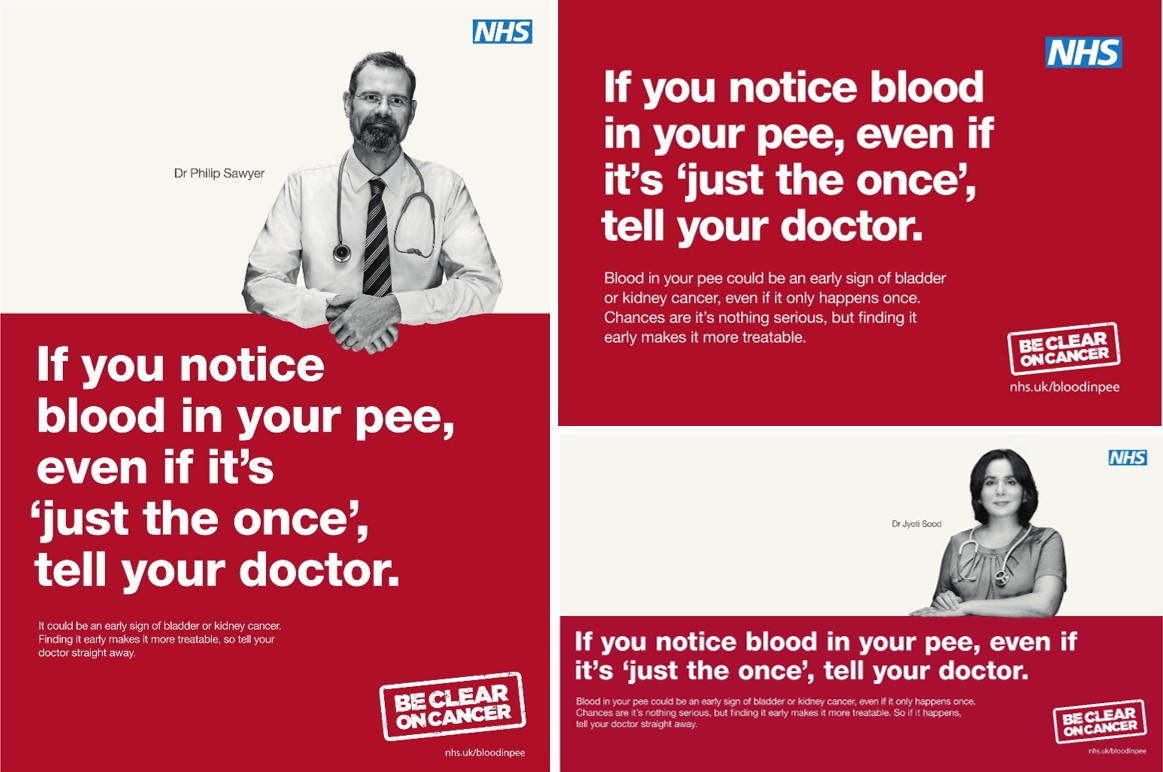 \| \| --- \| |
| \| SHOW OOH \| \| --- \| |
| \| Normal \| \| --- \| |
| \| 1 \| Yes, on a poster outside (such as a billboard or bus stop) \| \| --- \| --- \| \| 2 \| Yes, on a poster in a GP surgery \| \| 3 \| Yes, on a poster in a pharmacy \| \| 4 \| Yes, in a public / workplace toilet \| \| 5 \| Yes, as a leaflet [ASK IN 75+ ONLINE OMNI ONLY] \| \| 6 \| Yes, seen these or something like there but not sure where**Position fixed *Exclusive* \| \| 7 \| No, seen none of these ads **Position fixed *Exclusive* \| \| 999 \| Don't know **Position fixed *Exclusive* \| |

| \| Q042 - SEEN_LEAFLET: \| Single coded \| \| --- \| --- \| |
| --- | --- | --- |
| \| Not back \| \| --- \| |
| \| Have you seen this leaflet in the last couple of months?  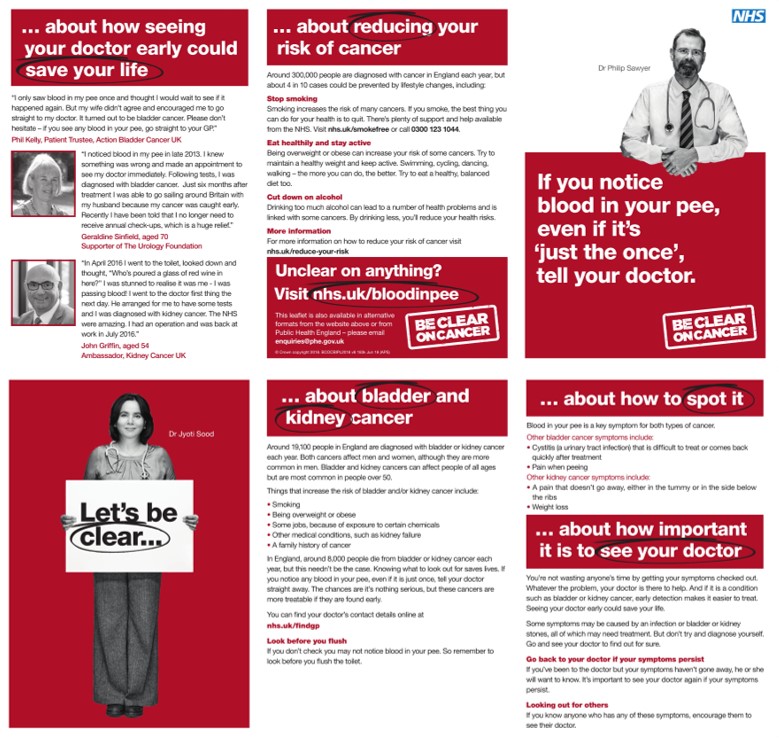 \| \| --- \| |
| \| SHOW LEAFLET \| \| --- \| |
| \| Normal \| \| --- \| |
| \| 1 \| Yes \| \| --- \| --- \| \| 2 \| No \| \| 999 \| Don't know \| |

| \| Q034 - MAIN_MESSAGE: \| Open \| \| --- \| --- \| |
| --- | --- | --- |
| \| Not back \| \| --- \| |
| \| What do you think was the main message of all the adverts you have just seen? \| \| --- \| |
| \|  \| \| --- \| |
| \| 999 \| Don't know **Position fixed *Exclusive* \| \| --- \| --- \| |

| \| Q035 - ADS_ATTS: \| Matrix \| \| --- \| --- \| |
| --- | --- | --- |
| \| Not back \| Number of rows: 9 \| Number of columns: 6 \| \| --- \| |
| \| You are now going to see some statements about the advertising you have seen and heard. Thinking about all of this advertising, please state to what extent you agree or disagree with each statement. \| \| --- \| |
| \| RANDOMISE STATEMENT ORDER \| \| --- \| |
| \| Rows: Normal \| Columns: Normal \| \| --- \| |
| \| Rendered as Dynamic Grid \| \| --- \| |
| \|  \| Agree strongly \| Agree slightly \| Neither agree nor disagree \| Disagree slightly \| Disagree strongly \| Don't know \| \| --- \| --- \| --- \| --- \| --- \| --- \| --- \| \| The advertising is relevant to me \| 🔾 \| 🔾 \| 🔾 \| 🔾 \| 🔾 \| 🔾 \| \| The advertising told me something new \| 🔾 \| 🔾 \| 🔾 \| 🔾 \| 🔾 \| 🔾 \| \| This advertising stands out from other advertising \| 🔾 \| 🔾 \| 🔾 \| 🔾 \| 🔾 \| 🔾 \| \| This advertising is clear and easy to understand \| 🔾 \| 🔾 \| 🔾 \| 🔾 \| 🔾 \| 🔾 \| \| It is important that adverts like this are shown \| 🔾 \| 🔾 \| 🔾 \| 🔾 \| 🔾 \| 🔾 \| \| This advertising would make me more likely to go to my GP if I had any of these symptoms \| 🔾 \| 🔾 \| 🔾 \| 🔾 \| 🔾 \| 🔾 \| \| This advertising made me more likely to check the colour of my pee \| 🔾 \| 🔾 \| 🔾 \| 🔾 \| 🔾 \| 🔾 \| \| I am fed up of seeing this type of advertising about cancer \| 🔾 \| 🔾 \| 🔾 \| 🔾 \| 🔾 \| 🔾 \| \| I trust the information given by these ads \| 🔾 \| 🔾 \| 🔾 \| 🔾 \| 🔾 \| 🔾 \| |

| \| ASK IF **Q31**,1,2,3,4,5 OR **Q32**,1,2,3,4,5 OR **Q33A**,1 OR **Q40**,1,2, **Q39**,1,2,3,4,5,6, **Q42,**1 \| \| --- \| |
| --- | --- |
| \| Q036 - ACTION_TAKEN: \| Multi coded \| \| --- \| --- \| |
| \| Not back \| Min = 1 \| \| --- \| |
| \| As a direct result of seeing or hearing any of these ads you have just seen, did you do any of the following... \| \| --- \| |
| \| Normal \| \| --- \| |
| \| 1 \| Checked the colour of my pee \| \| --- \| --- \| \| 2 \| Thought about making an appointment to talk to my GP \| \| 3 \| Made an appointment to talk to my GP \| \| 4 \| Talked to another healthcare professional \| \| 5 \| Talked to friends or family members about symptoms of my own \| \| 6 \| Talked to friends or family members to advise them about the information in these ads \| \| 7 \| Visited an NHS website for further advice or information \| \| 8 \| Called NHS 111 for further advice or information \| \| 9 \| Nothing **Exclusive* \| \| 10 \| Something else **Open* \| \| 11 \| Don't know \| |

| \| Q037 - SEEN_BCOC: \| Multi coded \| \| --- \| --- \| |
| --- | --- | --- |
| \| Not back \| Min = 1 \| \| --- \| |
| \| As well as the advertising, have you seen or heard any of the following Be Clear on Cancer activities about blood in pee? \| \| --- \| |
| \| RANDOMISE \| \| --- \| |
| \| Normal \| \| --- \| |
| \| 1 \| Article in a newspaper \| \| --- \| --- \| \| 2 \| Article online / social media \| \| 3 \| Article in a magazine \| \| 4 \| News feature on TV \| \| 5 \| News feature on radio \| \| 6 \| Other **Open* \| \| 7 \| I have not seen any of these \| \| 8 \| Don't know \| |

| \| \| B006 - B006: CAMPAIGN RECOGNITION \| End block \| \| --- \| --- \| \| \| --- \| --- \| --- \| |
| --- | --- | --- | --- |

| \| \| B007 - B007: ADDITIONAL DEMOGRAPHICS \| Begin block \| \| --- \| --- \| \| \| --- \| --- \| --- \| |
| --- | --- | --- | --- |

| \| Q029 - ETHNICITY: \| Single coded \| \| --- \| --- \| |
| --- | --- | --- |
| \| Not back \| \| --- \| |
| \| Which of these best describes your ethnic group? \| \| --- \| |
| \| Please could you group the answers as follows, with titles above each grouping.  WHITE (Title above 1,2,3)  MIXED (Title above 4,5,6,7)  ASIAN OR ASIAN BRITISH (8,9,10)  BLACK OR BLACK BRITISH (11,12,13)  CHINESE/ OTHER (14,15)  16 by its self \| \| --- \| |
| \| Normal \| \| --- \| |
| \| 1 \| White British \| \| --- \| --- \| \| 2 \| White Irish \| \| 3 \| Any other white background \| \| 4 \| Mixed - white & black Caribbean \| \| 5 \| Mixed - white & black African \| \| 6 \| Mixed - white & Asian \| \| 7 \| Any other mixed background \| \| 8 \| Indian \| \| 9 \| Pakistani \| \| 10 \| Bangladeshi \| \| 11 \| Black Caribbean \| \| 12 \| Black African \| \| 13 \| Any other Black background \| \| 14 \| Chinese \| \| 15 \| Other \| \| 16 \| Prefer not to say \| |

| \| Q030 - MARITAL_STATUS: \| Single coded \| \| --- \| --- \| |
| --- | --- | --- |
| \| Not back \| \| --- \| |
| \| What is your marital status? \| \| --- \| |
| \| Normal \| \| --- \| |
| \| 1 \| Married / living as married \| \| --- \| --- \| \| 2 \| Single \| \| 3 \| Widowed / divorced / separated \| |

| \| \| B007 - B007: ADDITIONAL DEMOGRAPHICS \| End block \| \| --- \| --- \| \| \| --- \| --- \| --- \| |
| --- | --- | --- | --- |
